# Unique starch biosynthesis pathways in wild rice revealed by multi‐omics analyses

**DOI:** 10.1111/pbi.70021

**Published:** 2025-03-27

**Authors:** Nurmansyah, Agnelo Furtado, Pauline Okemo, Robert J. Henry

**Affiliations:** ^1^ Queensland Alliance for Agriculture and Food Innovation University of Queensland Brisbane Queensland Australia; ^2^ ARC Centre for Plant Success in Nature and Agriculture University of Queensland Brisbane Queensland Australia; ^3^ Department of Agronomy, Faculty of Agriculture Universitas Gadjah Mada Yogyakarta Indonesia

**Keywords:** wild rice, eating and cooking quality, multi‐omics analyses, slowly digested starch

## Abstract

Australian wild rice species (AWS) possess unique starch properties characterized by a slow digestibility rate. However, the genomic and transcriptomic variations of starch‐synthesis‐related genes (SSRGs) influencing starch physiochemical properties in AWS remain unclear. Here, we report comparative analyses of 72 SSRGs in wild species, including two AWS (*O. meridionalis* and Australian populations of *O. rufipogon*) and the domesticated rice gene pool. Our findings reveal that most SSRGs are more actively expressed in the early stages of seed development. Transcriptome analysis identified differential splicing patterns, with the starch synthesis pathways in Nipponbare and *O. rufipogon* being more similar than those in *O. meridionalis*. Three essential starch genes, *GBSSI, SSIIa* and *BEIIb*, were more active and had higher expression in AWS compared to Nipponbare, explaining the higher amylose content, gelatinization temperature, soft gel consistency and high retrogradation in the wild rice. Comparative genomics indicated that Asian domesticated rice evolved from a single ancestral allele of *GBSSI* (*Wx* ^*lv*^) and *SSIIa* (*ALK* ^*c*^), but two *BEIIb* alleles originated from *O. rufipogon* and *O. nivara*, the two wild rice species that are considered progenitors of Asian domesticated rice. Additionally, higher expressions of *GBSSI*, *BEI* and *SSIIIa* in *O. meridionalis* contribute to a slower starch digestibility rate, making its haplotypes valuable for breeding to develop slowly digested starch cultivars. These findings not only provide insight into the evolution of starch gene synthesis during domestication but also pave the way for unlocking desirable gene haplotypes of wild rice to improve starch quality in cultivated rice.

## Introduction

Rice is one of the leading staple crops, consumed by over half of the global population. Starch is the most abundant component in rice grain. It comprises up to 90% of the dry weight of white rice and 72%–82% of brown rice (Bao, 2019). Although brown rice is the highest carbohydrate source per edible portion among cereals, it contains the lowest dietary fibre (DF), with only 3.5 g/100 g compared with 16.6 g/100 g in sorghum grain (USDA, 2016). The polishing of white rice removes the outer layer (rice bran) and embryo where most of the DF is found, and consequently, white rice contains even lower DF with only 0.7–2.7 g /100 g (Juliano, 2016). The ratio of carbohydrates to DF and the amount of resistant starch are two main components of the carbohydrate quality index. Foods with more DF, slowly digested starches and resistant starch are considered to have a higher carbohydrate quality (Seal *et al*., 2021).

Many approaches have been used to enhance rice carbohydrate quality, including the alteration of various starch genes and the combination of different alleles of starch genes. The changes have been performed by knocking out or downregulating several starch genes, such as starch synthase IIIa (*SSIIIa*) and *branching enzyme IIb* (*BEIIb*). Knocking out *SSIIIa* in an indica rice background can increase the development of resistant starch type 5 (RS5). RS5 is an amylose and lipid complex that generates a helical structure, inhibiting amylase digestion (Shen *et al*., 2022). The lack of *SSIIIa* increases *granule‐bound starch synthase I* (*GBSSI*) expression, leading to an increase in the amount of amylose‐lipid complex and consequently RS5 (Zhou *et al*., 2016). Similarly, the loss of function of *BEIIb* can also increase resistant starch in indica and japonica rice cultivars. Knocking out *BEIIb* generates longer amylopectin chains and creates a type B crystalline structure, hindering the access of digestive enzymes to the glucose chains (Tsuiki *et al*., 2016; Yi *et al*., 2021). However, the absence of these genes also results in adverse consequences, including a yield penalty and deterioration of grain appearance quality (Ryoo *et al*., 2007; Butardo *et al*., 2011; Zhu *et al*., 2012; Sun *et al*., 2017; Baysal *et al*., 2020). Alternatively, the combination of different alleles of starch genes can be utilized to modify rice starch properties and elevate carbohydrate quality (Fujita *et al*., 2022). However, the lack of genetic diversity among domesticated rice cultivars poses a challenge in the development of rice cultivars with superior carbohydrate quality. Thus, unlocking novel haplotypes from wild rice has the potential to create new opportunities to enhance the genetic diversity in domesticated rice, and improve rice carbohydrate quality.

Australian wild rice species (AWS) are the most divergent species in the A genome clade that includes domesticated rice. These species are mainly found in northern Australia and exhibit desirable traits, such as disease resistance, large grain size and unique starch properties (Henry, 2019). AWS was found to have a slower *in vitro* digestion rate compared to some cultivated rice cultivars (Zhao *et al*., 2021). The slow digestion rate of AWS has been attributed to their unique starch properties. Compared to domesticated rice cultivars, AWS starches had higher amylose content, more amylose shorter chains, fewer amylopectin short chains and more amylopectin long chains. These starch structure properties of AWS resulted in a higher gelatinization temperature and gelatinization enthalpy, as well as a slower in‐vitro digestion rate (Tikapunya *et al*., 2017; Zhao *et al*., 2021). Moreover, AWS had similar textural characteristics to some domesticated rice cultivars, as well as acceptable sensory characteristics. Consequently, AWS could serve as a valuable source of nutritionally beneficial yet palatable starch that is digested slowly (Tikapunya *et al*., 2018; Zhao *et al*., 2021, 2022).

Variation in starch‐synthesis‐related genes (SSRGs) may influence their expression and determine starch properties. Importantly, the molecular structural properties of rice starch have a significant role in determining eating quality, including gel consistency and gelatinization temperature (Tao *et al*., 2019; Tian *et al*., 2009; Wang *et al*., 2017; Zhu *et al*., 2023) as well as in vitro digestibility (Li *et al*., 2021). The starch properties of AWS have been extensively studied (Kasem *et al*., 2014; Tikapunya *et al*., 2017; Tikapunya *et al*., 2018; Zhao *et al*., 2021; Zhao *et al*., 2022). However, comparative studies of genomic and transcriptomic variations of SSRGs in AWS are limited. Therefore, deciphering the genomic and transcriptomic profiles of AWS will be the key to uncovering desirable starch alleles. In the present study, 72 genes known to regulate starch biosynthesis in rice were extensively studied to understand the genetic variability and gene expression dynamics within two AWS (*Oryza meridionalis* and Australian populations of *O. rufipogon*) and the reference japonica genotype of *O. sativa*, Nipponbare. The diversity of these genes in the domesticated rice gene pool was also analysed. Comprehensive analyses of genetic polymorphisms and gene expression profiling of SSRGs will help to understand the different molecular mechanisms involved in starch biosynthesis in wild and domesticated rice. Furthermore, the information will be helpful in identifying the most desired haplotypes for grain quality improvement, especially for developing slowly digestible starch cultivars.

## Results

### Starch synthesis pathway of Nipponbare and *O. rufipogon* is more comparable than that of *O. meridionalis*

The expression profiles of 72 SSRGs differed across genotypes and seed‐developing stages. However, the gene expression levels could be divided into four groups: high, upper‐moderate, lower‐moderate and low expression levels, based on hierarchical clustering (Figure S1; Table S3). To decipher differentially expressed genes (DEGs) of 72 SSRGs between different stages of seed development, two comparison groups were analysed: 15 DPA against 5 DPA (15 DPA/5 DPA) and 25 DPA against 15 DPA (25 DPA/15 DPA) across three genotypes. Most DEGs were downregulated as the seed matured in the three genotypes, suggesting that starch synthesis genes are more actively synthesized in the early stages of seed development (Figure S2a, b). Another observation when comparing DEGs through seed development was that Nipponbare and *O. rufipogon* shared more similarities than Nipponbare and *O. meridionalis* or *O. rufipogon* and *O. meridionalis*. Nipponbare and *O. rufipogon* have 18 and 22 similar DEGs in 15 DPA/5 DPA and 25 DPA/15 DPA, respectively. On the contrary, a total of 7 and 13 DEGs were detected between Nipponbare and *O. meridionalis* in 15 DPA/5 DPA and 25 DPA/15 DPA, respectively (Figure S2c, d). The findings indicate that the starch synthesis pathway between Nipponbare and *O. rufipogon* is more comparable than that between Nipponbare and *O. meridionalis* or *O. rufipogon* and *O. meridionalis*.

### Expressions of GBSSI, BEIIb and pGlcT are consistently upregulated, differentiating AWS from Nipponbare

To assess the difference in gene expression between AWS and Nipponbare during seed development, DEGs were analysed. The total number of DEGs between *O. rufipogon* and Nipponbare was 31, 8 and 19 genes in 5 DPA, 15 DPA and 25 DPA, respectively (Figure S3a). The majority of DEGs were upregulated at all development stages. A total of 37 out of 72 SSRGs were differentially expressed throughout different developmental stages (Figure S3b). Of these, six genes consistently showed differential expression: *GBSSI, BEIIb, BT1, pGlcT, SSI and OsEBP89* (Figure S3c). In the comparison between *O. meridionalis* and Nipponbare, the total number of DEGs was 30, 14 and 17 genes at 5 DPA, 15 DPA and 25 DPA, respectively (Figure S3d). The majority of DEGs were upregulated at all development stages. A total of 39 SSRGs were differentially expressed throughout development (Figure S3e). Among them, seven genes consistently showed differential expressions: *GBSSI, BEIIb, BT1, pGlcT, OsSUS1, ISA2, OsPPDKB and OsNAC26* (Figure S3f).

**Figure 1:**
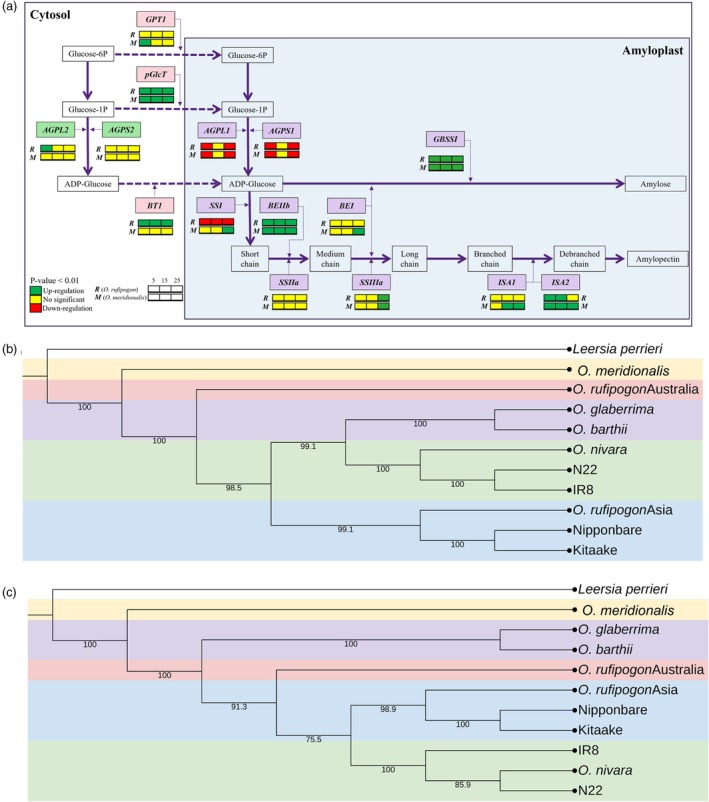
Starch synthesis pathways and phylogenetic relationships. (a) Temporal differential expression pattern of starch genes in developing seeds. The colour bars showing DESeq2 results consist of two rows and three columns. The first row, labelled R, represents differentially expressed genes (DEGs) between *O. rufipogon* and Nipponbare. The second row, labelled M, represents DEGs between *O. meridionalis* and Nipponbare. The columns correspond to three stages of seed development (from left to right): 5 days post‐anthesis (DPA), 15 DPA, 25 DPA. Colour coding indicates gene expression changes in wild rice compared to Nipponbare: green for up‐regulated genes, red for down‐regulated genes and yellow for non‐significant DEGs. (b) Gene sequence‐based phylogenetic tree of wild and domesticated rice species based upon 19 starch genes. (c) CDS‐based phylogenetic tree.

The key differences in the starch biosynthesis pathways of Australian wild rice compared to Nipponbare are shown in Figure 1a. The figure illustrates different gene expressions between AWS and Nipponbare at three seed development stages. Compared to Nipponbare, AWS had different expression profiles for most starch genes except *AGPS2* and *SSIIa*. In terms of the SS and BE genes, AWS had more active *GBSSI, BEIIb* and *SSIIIa* compared to Nipponbare. However, *O. meridionalis* had more active *BEI* and *SSI* than *O. rufipogon*. Taken together, *GBSSI, BEIIb and pGlcT* are consistently upregulated in Australian wild rice compared to Nipponbare, highlighting key differences in starch biosynthesis pathways during seed development.

### *O. meridionalis* had distinct starch genes compared to cultivated and other wild rice relatives

Phylogenetic trees based on gene (genomic) and coding sequences (CDS) of 19 key starch genes were constructed. The gene sequence‐based trees were employed to capture evolutionary events that occur in the entire set of starch genes. The gene sequence‐based tree revealed that *O. meridionalis* and the Australian *O. rufipogo*n were separated from Asian and African rice species (Figure 1b). The Asian rice species were divided into two clades. The first clade comprises *O. nivara* and two types of Asian domesticated rice from the two subpopulations: IR8 from Indica type and N22 from Aus type. The second clade consists of *O. rufipogon* Asia and two Japonica type, Nipponbare and Kitaake. The African wild and domesticated rice, *O. bathii* and *O. glaberrima*, were grouped into the same clade. Although the geographical distribution of AWS is closer to Asian rice, the phylogenetic tree reveals that Asian rice is genetically more closely related to African rice than AWS.

The CDS‐based tree emphasizes the evolutionary changes that directly influence protein‐coding regions. The CDS‐based tree showed a shift between African rice and the Australian *O. rufipogon* (Figure 1c). The tree indicates a closer relationship between the Australian *O. rufipogon* and Asian rice. Whereas African rice was grouped outside the Asian clades. This finding indicated that the starch biosynthesis of the Australian *O. rufipogon* was closely related to that of Asian rice, whereas *O. meridionalis* exhibits the most distinct starch biosynthesis compared to other *Oryza* species studied.

### *O. meridionalis* had a greater genetic variance from Nipponbare than *O. rufipogon*

Gene structural analysis was performed on the 72 SSRGs. The gene length varied from 627 bp for *OsMADS78* to 16 567 bp for*OMADS14*, while the CDS length ranged from 627 bp for *OsMADS78* to 5397 bp for *SSIIIa*. Pairwise alignment was utilized to identify variation within gene structures (5'UTR, exons, introns and 3'UTR) in two comparison groups (Nipponbare vs. *O. rufipogon* and Nipponbare vs. *O. meridionalis*). The CDS similarity between Nipponbare and *O. rufipogon* ranged from 96.3% to 100%, whereas the CDS similarity between Nipponbare and *O. meridionalis* ranged from 86.6% to 99.9%. Moreover, a total of 10 genes had identical CDS sequences between Nipponbare and *O. rufipogon*, while all genes have distinct CDS sequences between Nipponbare and *O. meridionalis*.

To determine synonymous and non‐synonymous substitutions, SNP and indel were identified throughout each gene's CDS. A total of 279 SNPs were detected across 72 genes between Nipponbare and *O. rufipogon*, whereas the number of SNPs observed between Nipponbare and *O. meridionalis* was four times higher (1158 SNPs) than that between Nipponbare and *O. rufipogon*. Although many SNPs were detected, only half of them (143 SNPs) were revealed to indicate non‐synonymous substitutions between Nipponbare and *O. rufipogon*. While only 40% of the detected SNPs altered amino acids between Nipponbare and *O. meridionalis*. The number of indels identified was lower than the number of SNPs between the two comparison groups. A total of 143 indels were found between Nipponbare and *O. rufipogon*, while the number of indels between Nipponbare and *O. meridionalis* was three times higher (458) than that between Nipponbare and *O. rufipogon* (Table 1).

**Table 1:** Structural variation of 72 genes between Nipponbare and AWS

No	Gene name	Gene Length (bp)			CDS Length (bp)			CDS similarity (%)		Nipponbare vs. *O. rufipogon*			Nipponbare vs. *O. meridionalis*				
										Exon		Amino acid		Exon		Amino acid	
		Nip	*O. ruf*	*O. mer*	Nip	*O. ruf*	*O. mer*	Nip vs *O. ruf*	Nip vs *O. mer*	SNP	Indel	Non‐Syn Subs	Indel	SNP	Indel	Non‐Syn Subs	Indel
1	*AGPL1*	5484	5445	5414	1560	1560	1560	100	99.1	0	0	0	0	14	0	5	0
2	*AGPL2*	8079	8107	8696	1557	1557	1557	99.9	99.9	2	0	0	0	2	0	0	0
3	*AGPL3*	5201	4732	4663	1536	1536	1527	100	97.6	0	0	0	0	22	15	6	5
4	*AGPL4*	6159	6158	6169	1530	1530	1530	99.9	99.3	2	0	1	0	10	0	1	0
5	*AGPS1*	4380	4391	4378	1503	1518	1509	97.8	99.3	19	15	6	5	5	6	4	2
6	*AGPS2*	6195	6227	6299	1440	1440	1440	99.7	98.4	4	0	0	0	23	0	2	0
7	*GBSSI*	5032	5022	5036	1830	1830	1830	99.6	99.6	8	0	2	0	8	0	2	0
8	*GBSSII*	4214	4216	4218	1827	1827	1827	99.2	99.2	15	0	12	0	14	0	8	0
9	*SSI*	7513	7508	7508	1926	1926	1926	99.9	99.9	1	0	0	0	1	0	0	0
10	*SSIIa*	4905	4907	4877	2433	2433	2415	99.7	96.9	8	0	5	0	45	30	15	10
11	*SSIIb*	4513	4518	4484	2085	2085	2079	99.8	97.7	5	0	5	0	37	10	22	2
12	*SSIIc*	7833	7827	7827	2250	2250	2253	99.8	98.3	5	0	2	0	27	11	15	1
13	*SSIIIa*	11 172	11 161	10 870	5367	5367	5397	99.9	98.7	7	0	3	0	39	30	19	10
14	*SSIIIb*	7782	7796	8530	3651	3651	3666	99.8	97.9	8	0	6	0	61	15	25	5
15	*SSVIa*	8998	9000	9019	2919	2919	2919	99.8	99.7	6	0	4	0	10	0	6	0
16	*SSVIb*	8172	8166	8030	2748	2748	2757	99.9	96.6	3	0	2	0	11	9	3	3
17	*BEI*	8426	7328	8260	2463	2463	2463	100	99.8	0	0	0	0	6	0	4	0
18	*BEIIa*	3250	3247	3220	1032	1026	1032	100	98.5	0	0	0	0	9	6	4	2
19	*BEIIb*	11 338	11 342	11 726	2478	2478	2475	99.8	99.3	5	0	1	0	14	3	3	1
20	*ISA1*	6920	6916	6893	2412	2412	2403	99.9	99.2	3	0	2	0	11	9	6	3
21	*ISA2*	2548	2539	2539	2403	2394	2394	99.5	99.5	4	9	3	3	4	9	3	3
22	*ISA3*	11 326	11 348	9987	2349	2349	2352	99.9	99.1	2	0	1	0	17	3	10	1
23	*DPE1*	4431	4430	4467	1785	1785	1800	99.8	97.9	4	0	4	0	21	15	9	5
24	*DPE2*	7433	7435	7436	2481	2481	2481	99.9	99.5	3	0	1	0	13	0	3	0
25	*BT1*	2185	2187	2291	1278	1278	1278	99.8	96.2	3	0	2	0	16	33	9	11
26	*GPT1*	3920	3921	3900	1164	1164	1164	99.8	98.7	2	0	0	0	15	0	2	0
27	*GPT2*	1951	1953	1952	828	828	828	99.9	99.3	1	0	1	0	6	0	0	0
28	*pGlcT*	5278	5259	5217	1680	1680	1680	99.9	99.2	2	0	2	0	14	0	5	0
29	*PHO1*	7131	7132	7143	2937	2949	2949	99.4	99.5	3	12	3	4	2	12	3	4
30	*PHO2*	6158	6172	6172	2526	2526	2526	99.8	99.8	5	0	2	0	5	0	2	0
31	*OsSUS1*	4811	4811	5003	2451	2451	2451	99.7	99.2	7	0	2	0	19	0	0	0
32	*OsSUS2*	5882	5882	5798	2427	2427	2427	99.8	99.1	6	0	0	0	23	0	3	0
33	*OsSUS3*	5494	5491	5449	2451	2451	2442	99.9	98.5	2	0	2	0	28	9	3	3
34	*NF‐YB1*	1004	1003	1008	561	561	555	99.3	96.3	4	0	2	0	15	6	5	2
35	*OsPPDKB*	7873	7886	7849	2649	2649	2649	99.9	99.9	2	0	0	0	1	0	0	0
36	*OsSK41*	6026	6020	5886	1275	1275	1275	99.8	99.3	3	0	1	0	9	0	0	0
37	*pPGM*	7394	7330	7523	1830	1830	1833	99.9	99.0	1	0	0	0	16	3	4	1
38	*GIF1*	4751	4777	4684	1797	1797	1791	99.7	98.8	6	0	3	0	16	6	9	2
39	*GWC1*	4237	4236	4240	825	825	825	99.5	99.6	4	0	1	0	3	0	1	0
40	*Du1*	4574	4573	4512	3120	3120	3111	99.9	98.4	1	0	0	0	41	9	5	3
41	*FLO2*	13 006	13 061	12 553	5163	5163	5154	99.8	98.8	9	0	5	0	53	9	25	3
42	*FLO6*	4950	4931	5555	1590	1575	1590	99.8	95.6	3	0	0	0	32	39	18	13
43	*FLO7*	3343	3343	3613	1095	1095	1104	99.7	97.6	3	0	1	0	17	9	6	3
44	*FLO15*	6443	6444	6421	1053	1053	1053	99.7	98.8	3	0	1	0	13	0	1	0
45	*FLO16*	3935	3941	3823	999	999	999	100	98.7	0	0	0	0	13	0	0	0
46	*FLR1*	3169	3185	3156	2682	2682	2655	99.7	98.2	7	0	3	0	22	27	10	9
47	*FSE1*	10 308	10 131	10 166	2814	2814	2814	99.8	99.4	6	0	4	0	17	0	8	0
48	*OsbZIP33*	4360	4360	4363	1278	1272	1275	99.5	99.5	1	6	1	2	4	3	2	1
49	*OsbZIP58*	4482	4482	4366	1311	1311	1320	99.6	96.8	5	0	4	0	22	21	13	7
50	*OsbZIP76*	3971	3960	3690	1341	1338	1335	98.8	96.7	13	3	11	1	38	6	20	2
51	*OsCDPK1*	4430	4439	4431	1629	1629	1629	99.9	99.6	2	0	1	0	6	0	2	0
52	*OsDOF18*	1556	1556	1553	1092	1092	1098	99.6	96.2	4	0	2	0	24	18	13	6
53	*OsEBP89*	1883	1887	1953	981	981	996	99.3	94.3	7	0	1	0	30	27	18	9
54	*OsGBP*	3771	3783	3785	921	921	921	99.6	99.5	4	0	4	0	5	0	4	0
55	*OsMADS1*	8464	9197	8532	774	774	774	100	99.1	0	0	0	0	7	0	1	0
56	*OsMADS6*	7968	8040	7794	753	753	753	100	99.5	0	0	0	0	4	0	1	0
57	*OsMADS7*	4336	4324	4018	750	750	750	99.7	99.1	2	0	2	0	7	0	1	0
58	*OsMADS14*	9796	9797	16 567	741	741	741	100	99.5	0	0	0	0	4	0	1	0
59	*OsMADS29*	4006	4061	3270	783	783	780	100	97.0	0	0	0	0	9	15	0	5
60	*OsMADS78*	646	646	649	633	633	636	100	97.5	0	0	0	0	13	3	4	1
61	*OsMADS79*	627	627	627	627	627	627	99.7	99.8	2	0	0	0	2	0	0	0
62	*OsNAC20*	1761	1710	1853	963	954	1035	96.3	91.6	3	33	1	11	15	72	9	24
63	*OsNAC26*	1866	1858	1821	1002	999	990	99.4	98.1	3	3	1	1	7	12	3	4
64	*OsNAP*	2393	2393	2425	1179	1179	1185	99.3	97.9	8	0	6	0	19	6	8	2
65	*OsPK2*	4637	4924	4647	1737	1737	1743	99.7	86.6	5	0	0	0	18	6	3	2
66	*OsSGL*	1062	1074	1048	768	780	783	98.1	90.4	3	12	1	4	9	69	4	23
67	*OsSRT1*	12 342	12 370	12 290	1452	1452	1452	99.9	99.2	1	0	1	0	12	0	8	0
68	*PFP1*	5221	5221	5185	1704	1704	1704	99.8	98.7	4	0	0	0	22	0	4	0
69	*RPBF*	3418	3425	3442	1122	1125	1140	98.7	92.8	6	9	4	3	32	51	29	17
70	*RSR1*	3404	3418	3405	1539	1539	1542	99.7	96.9	4	0	3	0	24	27	11	9
71	*SSG6*	3730	3736	3733	1629	1629	1629	99.8	99.8	4	0	4	0	4	0	4	0
72	*UGP1*	5076	5076	5076	1410	1410	1410	99.9	99.9	1	0	1	0	1	0	1	0
Total										279	102	143	34	1158	669	458	219

Abbreviations: Nip, Nipponbare; *O. ruf*, *O. rufipogon*; *O. mer*, *O. meridionalis*.

The phylogenetic relationships among the three species were constructed using CDS and protein sequences of the 72 genes (Figure S4). The phylogenetic trees show that Nipponbare was in the same clade as *O. rufipogon* and apart from *O. meridionalis*. Taken together, variant analysis and phylogenetic relationships of Nipponbare and Australian wild species reveal that Nipponbare starch genes are more closely related to *O. rufipogon* than *O. meridionalis*. These results support the finding that the expression analysis of the 72 starch synthesis genes between Nipponbare and *O. rufipogon* is more comparable than that between Nipponbare and *O. meridionalis*.

### *O. rufipogon* and *O. meridionalis* possess similar GBSSI structures, resulting in a higher expression level compared to Nipponbare

**Figure 2:**
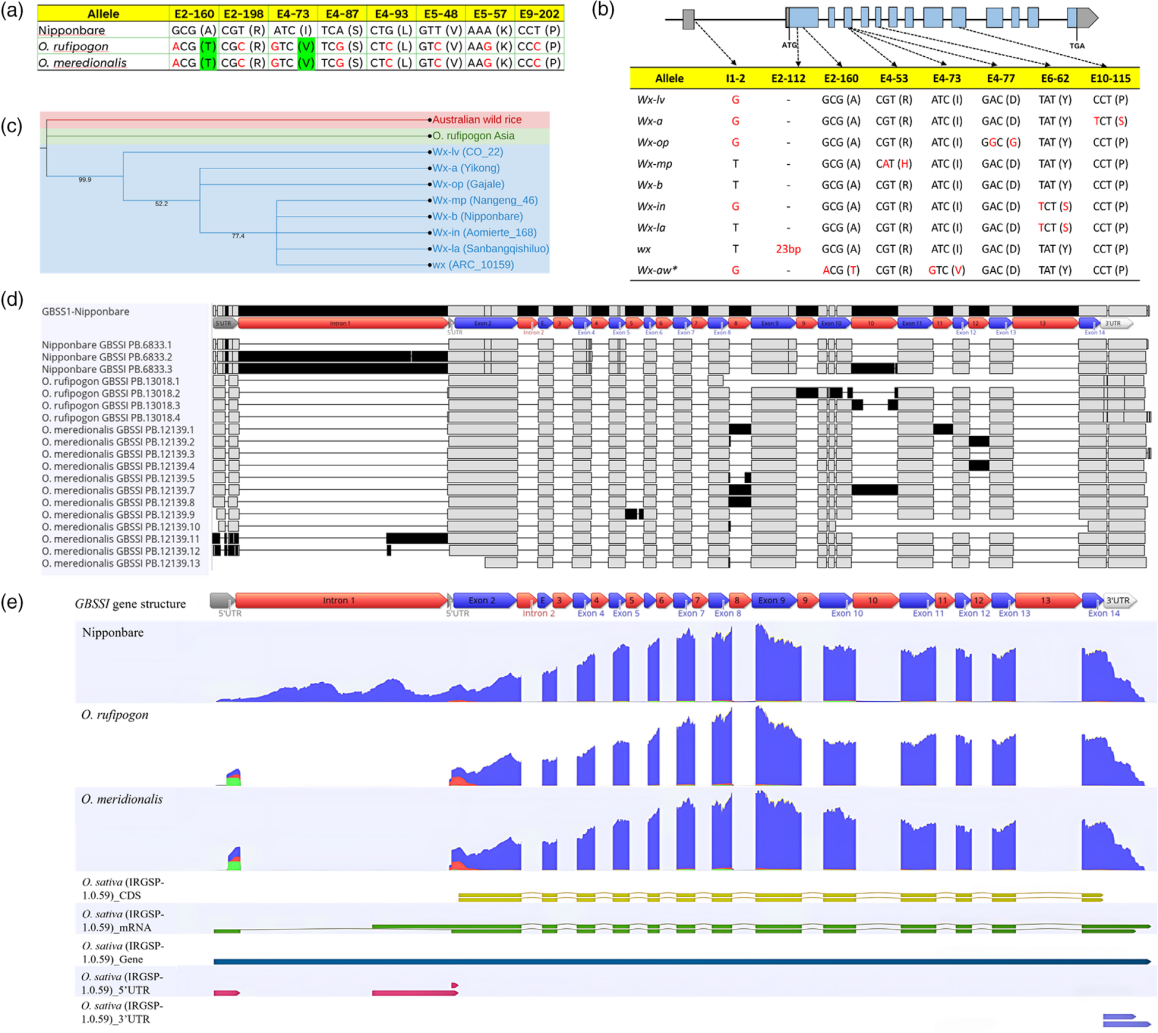
Haplotype and isoform analysis of *GBSSI*. (a) eight SNP positions and encoded amino acids (in parentheses) between Nipponbare and AWS. (b) The genomic structure of *GBSSI*, and the nucleotide sequences and encoded amino acids (in parentheses) in seven SNP sites between Asian haplotypes and AWS. (c) Phylogenetic tree of *GBSSI* on eight Asian haplotypes and AWS. The tree was generated using the Neighbour‐joining method with 1000 bootstrap replicates. *Wx‐aw is the assigned name for *GBSSI* of AWS. Red font letters indicate differences compared to reference *Wx‐b* Nipponbare. (d) Isoform diversity of *GBSSI* in three genotypes. The presence of intron retention in the isoform is represented by black bars in the intron region. (e) RNA‐seq read coverage indicated by blue, red and green colours. The first track illustrates RNA‐seq read coverage of *GBSSI* in Nipponbare, followed by *O. rufipogon* and *O. meridionalis* in the second and third tracks, respectively. The following tracks are CDS (yellow), mRNA (green), gene (blue), 5’UTR (red) and 3’UTR tracks (purple) based on IRGSP‐1.0 genome annotation.

The length of *GBSSI* varied across genotypes. Although the *GBSSI* length showed variation, the length of the CDS remained consistent across genotypes, suggesting the absence of any insertion or deletion that occurred in the CDS sequences. Interestingly, the CDS sequences of *O. rufipogon* and *O. meridionalis* were identical, with 99.6% similarity to Nipponbare. A total of eight SNPs were detected across four exons (Exons 2, 4, 5 and 9). Out of the eight SNPs, only two were identified to modify amino acid residues. The SNP E2‐160 (Exon 2 at nucleotide no. 160) substitution results in the conversion of alanine to threonine, whereas SNP E4‐73 causes a change from isoleucine to valine (Figure 2a).

We performed a haplotype analysis of the *GBSSI* gene in 4726 Asian rice accessions and then compared the results of this analysis with SNPs identified between Nipponbare and AWS. A total of 123 variants were observed across 4726 rice accessions, consisting of 101 SNPs and 22 indels. Of these, only 13 SNPs and one insertion were identified within exons. Furthermore, 4 out of 13 SNPs were functional resulting in amino acid substitutions (Table S4). The *GBSSI* gene has previously been classified into eight haplotypes based on six functional SNPs, including one SNPs in intron 1 (I1‐2) resulting in a splice donor variant (Zhou *et al*., 2021). Taken together, a total of 15 SNPs and one insertion were identified across 4726 Asian rice accessions with 5 functional SNPs and one insertion (Table S5). We compare these 15 SNPs and one insertion with the *GBSSI* of Australian wild rice. Interestingly, eight SNPs that were observed in the *GBSSI* of Australian wild rice were different from variants that were found in Asian rice. Moreover, the eight‐haplotype classification does not encompass two functional SNPs (SNP E2‐160 and SNP E4‐73) that have been identified in AWS.

We selected eight rice accessions that corresponded to the eight haplotypes and compared them with Australian wild rice. The *GBSSI* of AWS (*Wx‐aw*) was distinguished by three functional SNPs in comparison to the *GBSSI* of Nipponbare (*Wx‐b*) (Figure 2b). Furthermore, we constructed a phylogenetic tree of the eight *GBSSI* haplotypes of the Asian rice population, the *GBSSI* of AWS, and *O. rufipogon* from Asia (W1943). The phylogenetic tree distinguishes the *GBSSI* of AWS from the eight haplotypes of Asian rice (Figure 2c). These findings indicate that the *GBSSI* in AWS is novel relative to the Asian rice population.

### Iso‐seq analysis reveals *GBSSI* isoform diversity between AWS and Nipponbare

A SNP located in intron 1 at nucleotide no. 2 (I1‐2) has been reported as a genetic marker that divides rice cultivars with high and intermediate amylose content. The T to G mutation in I1‐2 had an impact on the quantity of mature *GBSSI* mRNA. The high amylose rice cultivars only possess a 2.3 kb mature mRNA, whereas the intermediate ones generate two transcripts, which are a 2.3 kb mature mRNA and a 3.3 kb pre‐mRNA (Cai *et al*., 1998; Zhang *et al*., 2019; Zhou *et al*., 2021). The 3.3 kb transcript is the 2.3 kb transcript containing a 1 kb intron 1. However, the 3.3 kb transcript of *GBSSI* remains unacknowledged in both the Rice Annotation Project Database (https://rapdb.dna.affrc.go.jp/) and the Rice Genome Annotation Project (http://rice.uga.edu/). Therefore, we performed an Iso‐seq analysis to explore transcript diversity between Nipponbare and AWS.

The Iso‐seq analysis demonstrates different *GBSSI* isoforms among the three genotypes (Figure 2d). In Nipponbare, three isoforms were discovered. The PB.6833.1 isoform incorporates all exons, 5’ UTR and 3’ UTR. The two other isoforms exhibited an alternative splicing event, involving intron retention. The PB.6833.2 isoform included intron 1, whereas PB.6833.3 contained introns 1 and 10. The *GBSSI* isoforms in AWS showed greater diversity compared to those of Nipponbare (Table S6). *O. rufipogon* possessed four isoforms, whereas *O. meridionalis* contained 12 isoforms. Interestingly, none of the isoforms in AWS carried all intron 1 sequences. To validate the findings of Iso‐seq analysis, we conducted a comparison of the read coverage of RNA‐seq data for *GBSSI* in the three genotypes (Figure 2e). The RNA‐seq reads aligned with intron 1 in the Nipponbare genome, as shown by the coverage graph in intron 1. On the contrary, a lower number of RNA‐seq reads were detected within intron I, as indicated by the absence of a coverage graph in the AWS genome. Altogether, these findings reveal that the lower amylose content in Nipponbare is attributed to reduced *GBSSI* expression and the formation of a 3.3 kb pre‐mRNA due to intron 1 retention, whereas AWS exhibits greater *GBSSI* isoform diversity without full intron 1 sequence.

### Higher expression of BEI in *O. Meridionalis* may result from indels in the promoter region

**Figure 3:**
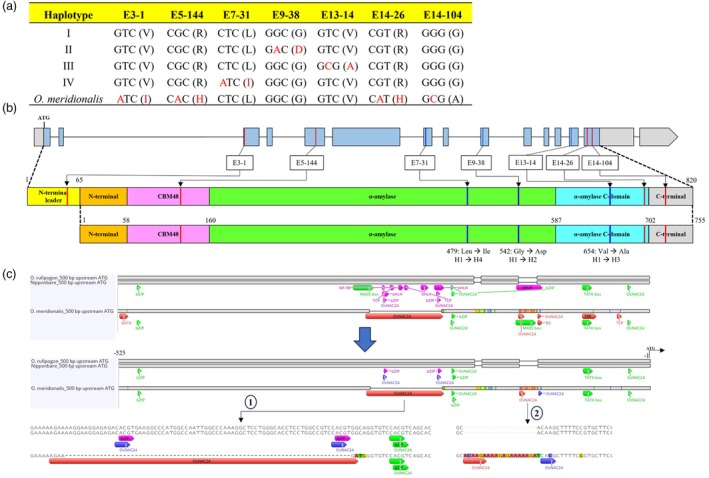
Genomic analysis of *BEI*. (a) Positions of seven SNPs and their corresponding encoded amino acids (in parentheses) among Asian haplotypes and *O. meridionalis*. (b) SNP positions mapped onto the genomic structure and schematic representation of the encoded *BEI* protein. Red lines indicate SNPs unique to *O. meridionalis* compared to Nipponbare, while blue lines represent SNPs found in Asian domesticated rice accessions. (c) Promoter architecture of *BEI* among three genotypes. Green annotation indicates similar motifs, including TATA box and MADS box. Red and pink annotations indicate specific motifs in *O. meridionalis* and Nipponbare, respectively.

The *branching enzyme I* (*BEI*) is located on chromosome 6 in the reverse position. The length of *BEI* differed across genotypes. The length of the CDS remained consistent across genotypes. Interestingly, the CDS sequences of *O. rufipogon* and Nipponbare were identical, whereas the CDS similarity of *O. meridionalis* compared to Nipponbare was 99.8%. A total of six SNPs were observed between *BEI* in Nipponbare and *O. meridionalis*, but only four SNPs were identified as functional SNPs (Table S7). A haplotype analysis of 4726 Asian rice accessions on *BEI* shows 94 variants, consisting of 80 SNPs and 14 indels. All indels were exclusively detected within intron regions. A total of 11 SNPs were found in exon regions, with six of them being classified as nonsynonymous SNPs. The six functional SNPs were, then, utilized to construct a haplotype network. Four haplotypes were generated (Figure S5; Table S8). More than 80% of rice varieties were classified in Haplotype 1, including Nipponbare. The four functional SNPs that were previously identified between Nipponbare and *O. meridionalis* were novel to that observed in Asian rice accessions. Thus, *BEI* in *O. meridionalis* was classified as a new haplotype (Figure 3a).

*BEI* gene is synthesized as a protein of 820 amino acid residues. The 820 residues were categorized into six domains, N‐terminal leader, N‐terminal, carbohydrate binding module 48 (CBM48), the central α‐amylase, α‐amylase C‐domain and C‐terminal. The N‐terminal leader, consisting of the first 65 residues, is subsequently processed to generate mature *BEI* protein (Noguchi *et al*., 2011). The amino acid substitutions resulting from SNPs detected in Asian rice and *O. meridionalis* were mapped to determine the specific protein domains that are affected. The four amino acid substitutions of *O. meridionalis* were located in the N‐terminal leader, CBM48, α‐amylase C‐domain and C‐terminal. The two amino acid substitutions of Asian rice were located within the central α‐amylase, whereas the other is located in the α‐amylase C‐domain (Figure 3b). SNP E7‐31 substitutes leucine for isoleucine and distinguishes between Haplotype 1 and 4. SNP E9‐39 alters glycine to aspartic acid, leading to the formation of Haplotype 2. SNP E13‐14 replaces valine with alanine and causes a transition from Haplotype 1 to Haplotype 3.

Promoter architecture analysis was conducted between Nipponbare and AWS to identify potential cis‐regulatory elements (CREs) associated with different *BEI* expressions. The expression level of *BEI* between Nipponbare and *O. rufipogon* is not significantly different across the three seed‐developing stages. In contrast, *BEI* expression in *O. meridionalis* is significantly different compared to Nipponbare and *O. rufipogon*, particularly at 25 DPA (Figure 1a). Interestingly, a region of 525 bp upstream of the start codon between Nipponbare and *O. rufipogon* is identical, whereas *O. meridionalis* exhibits multiple indels and SNPs. This region was then utilized to identify cis‐regulatory elements using PlantPAN 4.0 (www.plantpan.itps.ncku.edu.tw). Different CREs between *O. meridionalis* and Nipponbare are shown in Figure 3c. The *O. meridionalis* promoter exhibits several SNPs that result in the formation of some motifs, including TCP, TBP, B3 and GATA. The indels formed more bHLH, bZIP, NAC, NF‐YB and TCP motifs in Nipponbare, which replaced the *OsNAC24* motifs found in *O. meridionalis*. The expression level of putative genes associated with the CREs is available in Table S9. Among all of the putative genes associated with CREs, *OsNAC24* and *OsbZIP58* have been reported to bind with the *BEI* promoter (Jin *et al*., 2023; Wang *et al*., 2013). The *OsNAC24* protein has been identified to bind strongly to two specific motifs (AGAAGA and ACAAGA) and to bind weakly to the core NAC motif, CACG, in the *BEI* promoter (Jin *et al*., 2023). *O. meridionalis* possesses both specific motifs present in its *BEI* promoter, resulting from two indels. The first Indel involves an insertion in Nipponbare and *O. rufipogon* promoter, replacing a strong motif, AGAAGA, with two weaker motifs, CACG. The second involves a deletion of a strong motif, ACAAGA, in Nipponbare and *O. rufipogon*. In total, *O. meridionalis* had two strong motifs and three weak motifs, whereas Nipponbare and *O. rufipogon* had only four weak motifs. Moreover, the expression levels of *OsNAC24* and *OsbZIP58* in *O. meridionalis* are higher than that of Nipponbare and *O. rufipogon* (Table S9). These results indicate that the presence of *OsNAC24* motifs within the *BEI* promoter and higher expression of *OsNAC24* and *OsbZIP58* may lead to an increase in the expression level of *BEI* in *O. meridionalis*.

### *BEIIb* has two major haplotypes, derived from two wild ancestors

*BEIIb* is located on chromosome 2 in reverse orientation. Although the length of *BEIIb* in *O. meridionalis* is longer than that of Nipponbare and *O. rufipogon*, the CDS length of *BEIIb* in *O. meridionalis* is shorter (2475 bp) due to the presence of a 3 bp deletion. The CDS similarity of *O. rufipogon* and *O. meridionalis* compared to Nipponbare is 99.8% and 99.3%, respectively. A total of five SNPs were observed between *BEIIb* in Nipponbare and *O. rufipogon*, but only one SNP was identified to change amino acid residues. On the contrary, 13 SNPs and 3 bp deletion were detected on *BEIIb* of *O. meridionalis* compared to Nipponbare. Three out of 13 SNPs were indicated as functional SNPs.

**Figure 4:**
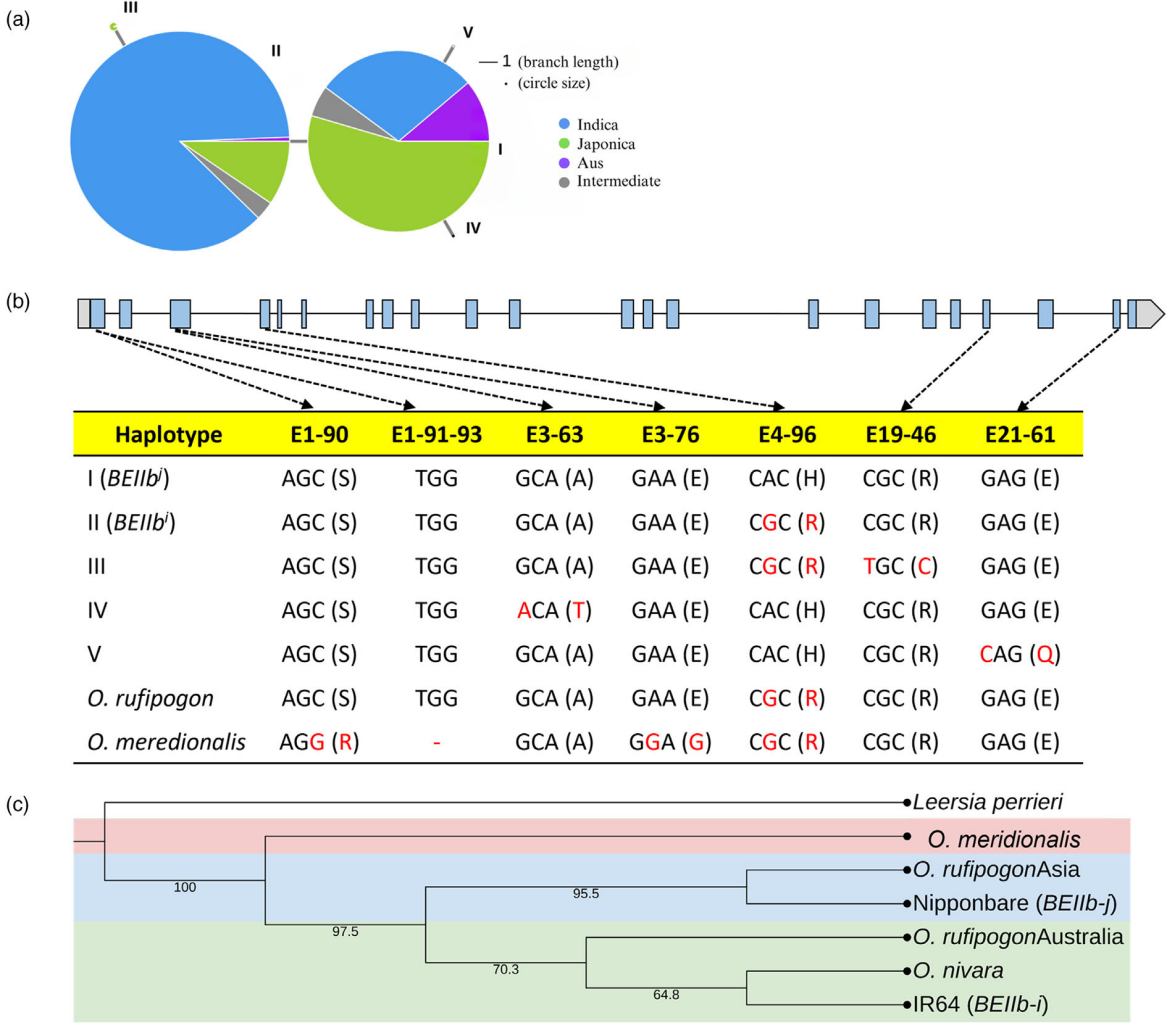
Haplotype analysis of *BEIIb*. (a) Haplotype network of *BEIIb* based on Asian rice accessions. (b) The genomic structure of *BEIIb*, and the nucleotide sequences and encoded amino acids (in parentheses) in seven SNP sites between Asian haplotypes and AWS. (c) Phylogenetic tree of *BEIIb* on different rice species. The tree was generated using the Neighbour‐joining method with 1000 bootstrap replicates.

A haplotype analysis of 4726 Asian rice accessions was conducted on the *BEIIb* gene to compare it with the *BEIIb* of AWS. A total of 133 variants were observed across Asian accessions, consisting of 109 SNPs and 24 indels. Among all variants, 53 are located within exons, consisting of 49 SNPs and 4 indels. Eleven out of 49 SNPs were identified as functional SNPs. Subsequently, the 11 SNPs are used to construct a haplotype network among Asian rice accessions. Five haplotypes were generated (Figure 4a, Table S10). Most Asian rice accessions (97%) resided in haplotypes I and II, with haplotype I mostly consisting of the Japonica type and haplotype II predominantly comprising the Indica type. Therefore, we annotate haplotype I as *BEIIb* ^*j*^ and haplotype II as *BEIIb* ^*i*^. The *BEIIb* haplotypes were constructed mainly due to four main SNPs, which were located in E3‐63 (Exon 3, nucleotide no. 63), E4‐96, E19‐46 and E21‐61. The *BEIIb* ^*j*^ and *BEIIb* ^*i*^ are differentiated by SNP E4‐96, which results in a substitution of histidine with arginine. Based on the haplotype classification, *BEIIb* of *O. rufipogon* was referred to as *BEIIb* ^*i*^, while *BEIIb* of *O. meridionalis* was distinct due to the presence of two unique SNPs (E1‐90 and E3‐76) and a 3 bp deletion (Figure 4b).

A phylogenetic tree was created among different rice species, including japonica rice Nipponbare (representing *BEIIb* ^*j*^), indica rice IR64 (representing *BEIIb* ^*i*^), Asian wild ancestors (*O. rufipogon* and *O. nivara*) and Australian wild rice (Figure 4c). *BEIIb* of *O. meridionalis* was outside the Asian rice clade, suggesting that it had a unique structure. In contrast, *BEIIb* of *O. rufipogon* Australia was grouped together with IR64 and *O. nivara*, conforming close relationship among those species regarding the *BEIIb* gene. Interestingly, the CDS of *BEIIb* from Nipponbare and Asian *O. rufipogon* were identical. Similarly, the CDS sequences of *BEIIb* in IR64 and *O. nivara* were the same. This finding suggests that *BEIIb* ^*j*^ originated from Asian *O. rufipogon*, whereas *BEIIb* ^*i*^ was derived from *O. nivara*.

### Different expressions of TFs results in varying SSI expressions

The expression of *starch synthase I* (*SSI*), *starch synthase II* (*SSIIa*) *and SSIIIa* varied among three genotypes. The expression of *SSI* in *O. rufipogon* is the lowest among the three genotypes, whereas the expression of *SSI* in *O. meridionalis* is higher than that of Nipponbare at 25 DPA. Although the expression of *SSI* varied between AWS compared to Nipponbare, the *SSI* gene structure between AWS is identical, with only one non‐functional SNP detected compared to Nipponbare's *SSI*, suggesting gene similarity across three genotypes. Promoter architecture analysis was conducted among three genotypes to identify potential CREs associated with different *SSI* expressions. Several indels and SNPs were detected within the 2.5 kb region upstream of the start codon among genotypes. Three multiple microsatellite (SSR) elements in the *SSI* gene have previously been reported to divide rice cultivars with different starch physicochemical properties. Three combinations of SSR were SSS‐A: (AC)_2_ …TCC(TC)_11_ …(TC)_5_C(ACC)_11_, SSS‐B: (AC)_3_…TCT(TC)_6_…(TC)_4_C(ACC)_9_ and SSS‐C: (AC)_3_…TCT(TC)_6_…(TC)_4_C(ACC)_8_ (Bao *et al*., 2002). SSS‐B contains one additional ACC element in comparison to SSS‐C. The gene structural analysis of the gene indicated that these SSR elements were located within 5’UTR. SSR polymorphism analysis showed that Nipponbare has eight copies of the ACC element, while AWS contains seven copies. On the contrary, IR64, an indica type, possesses nine copies (Figure S6a).

**Figure 5:**
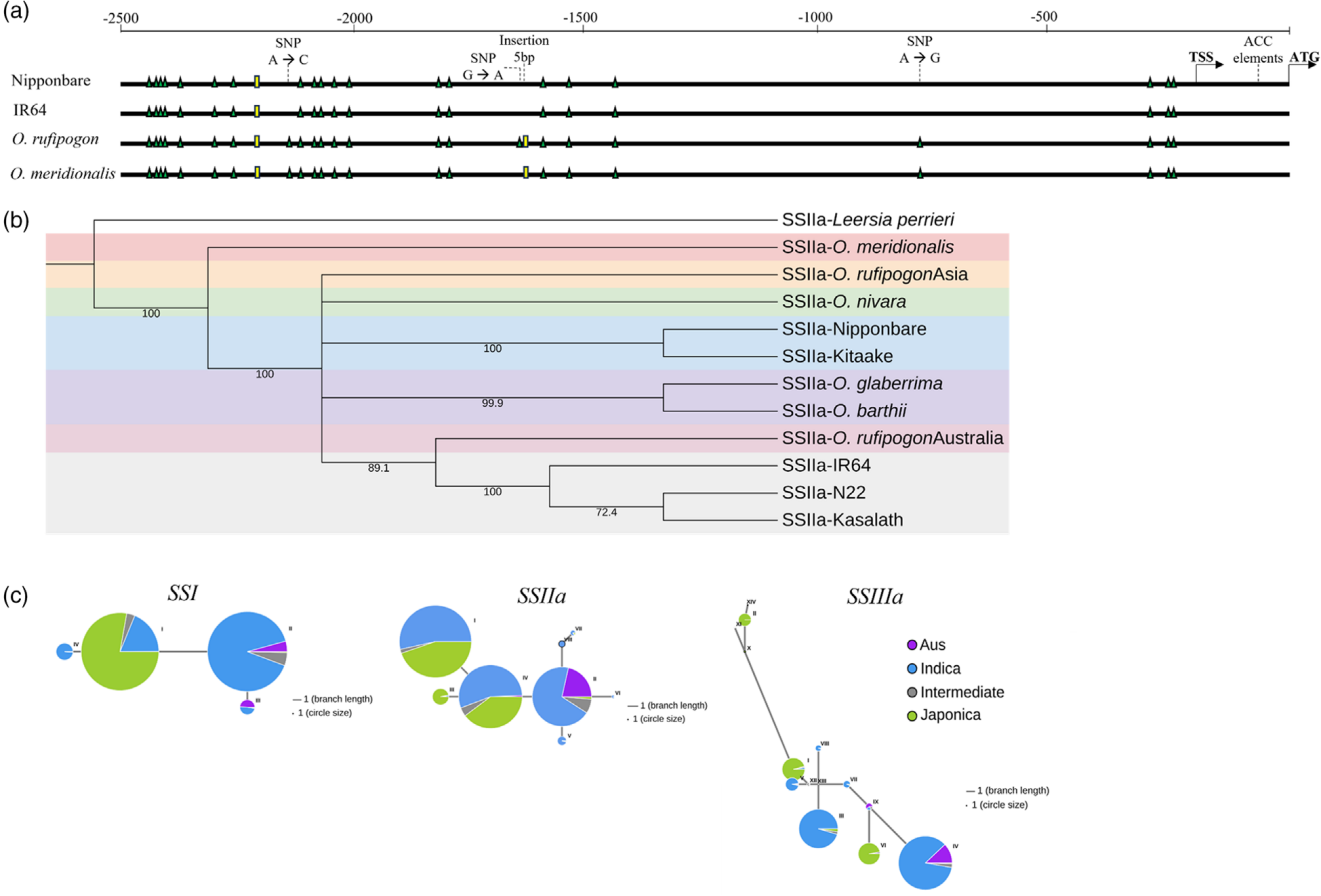
Analysis on three key soluble starch synthase genes. (a) Schematic representation of CRE motif distribution in the *SSI* promoter. The green triangle indicates the NAC motif, yellow rectangle indicates CCAAT‐box motif. (b) The phylogenetic tree of the *SSIIa* gene among Asian rice cultivars and wild relatives. (c) Haplotype network among three soluble starch synthase genes.

The promoter region of *SSI* is enriched with CACG motifs, which are the core binding motifs of NAC transcription factors (TFs). Several SNPs provide more CACG motifs in AWS compared to Nipponbare (Figure 5a). Among eight endosperm‐specific NAC TFs, *OsNAC20* and *OsNAC26* have been reported to regulate *SSI* expression (Wang *et al*., 2020). The expression levels of *OsNAC20* and *OsNAC26* varied across three genotypes. The expression of *OsNAC20* in Nipponbare is the highest at 5 and 15 DPA, whereas, at 25 DPA, *O. meridionalis* exhibited the highest *OsNAC20* expression. In contrast, the expression of *OsNAC26* in *O. meridionalis* is the highest across three developing stages (Figure S6b). In addition to the CACG motif, the *SSI* promoter region contains a CCAAT‐box motif, which serves as a binding site for nuclear factor‐Y (NF‐Y) TFs. Nipponbare possesses only one CCAAT‐box motif, whereas AWS contains two motifs, due to indels at −877 bp in the *SSI* promoter (Figure S6c). Among eight endosperm‐specific NF‐Y TFs, only *NF‐YA8* has been documented to influence *SSI* expression (Lu *et al*., 2024). *O. meridionalis* demonstrated the highest expression level of *NF‐YA8* across three genotypes, whereas *O. rufipogon* exhibited the lowest expression level (Figure S6d). Altogether, different expressions of TFs that bind to *SSI* promoters may result in different expression levels of *SSI* among the three genotypes.

### *ALK* ^*c*^ is the ancestral allele of *SSIIa*

The expressions of *SSIIa* exhibited no significant differences across the three genotypes. However, previous studies reported that three SNPs affected *SSIIa* activity (Nakamura *et al*., 2005). The three SNPs then further categorized *SSIIa* into three haplotypes, which are *ALK* ^*a*^, *ALK* ^*b*^ and *ALK* ^*c*^. *ALK* ^*c*^ is an active type of *SSIIa*, prevalent among indica rice cultivars. *ALK* ^*a*^ is a less active type of *SSIIa*, which is common to japonica rice cultivars. *ALK* ^*a*^ exhibited just 10% of the activity of *ALK* ^*c*^, whereas the activity of *ALK* ^*b*^ is intermediate between that of *ALK* ^*a*^ and *ALK* ^*c*^ (Chen *et al*., 2020; Nakamura *et al*., 2005). Based on this classification, Nipponbare is classified as *ALK* ^*a*^, whereas AWS is classified as *ALK* ^*c*^ (Table S11). Thus, although *SSIIa* expression levels were relatively similar across genotypes, the *SSIIa* activity was different between Nipponbare and AWS. Furthermore, analysis on various wild and cultivated rice varieties, including Asian varieties (Kitaake, IR64, Kasalath and N22) and their ancestors (*O. rufipogon* and *O. nivara*), as well as African rice (*O. glaberrima* and *O. barthii*), revealed that the majority exhibited *ALK* ^*c*^, with the exception of Kitaake, which possesses *ALK* ^*b*^ (Table S11). The phylogenetic tree of the *SSIIa* gene further illustrated the separation of japonica rice varieties, Nipponbare and Kitaake, from indica rice and its ancestor *O. rufipogon* Asia (Figure 5b). To explore the distribution of *SSIIa* haplotypes, a haplotype analysis on the three SNPs was performed on 4726 Asian rice accessions. The haplotype analysis revealed that 63.6% of rice varieties were classified as *ALK* ^*c*^, 30.8% as *ALK* ^*b*^ and the remaining 5.6% as *ALK* ^*a*^ (Figure S7). Collectively, these findings indicate that *ALK* ^*c*^ is the origin of the *SSIIa* gene, whereas *ALK* ^*a*^, found mostly in japonica rice, occurred due to a mutation of *ALK* ^*c*^.

### *SSIIIa* had a greater functional variant density

*SSIIIa* is the longest CDS sequence compared to other SSRGs. Compared to Nipponbare, *O. meridionalis* exhibited more variations than *O. rufipogon*, displaying 39 SNPs and 30 bp insertion within exon regions. Whereas *O. rufipogon* possessed seven SNPs compared to Nipponbare. The *SSIIIa* gene also generated greater variations within 4726 Asian rice accessions compared to *SSI* and *SSIIa*. A total of 33 nonsynonymous SNPs were identified within *SSIIIa* CDS across Asian cultivars, while only 10 and 13 nonsynonymous SNPs were found within *SSI* and *SSIIa*, respectively. Furthermore, a haplotype network analysis illustrated that *SSIIIa* had more haplotypes (14 haplotypes) than *SSI* (4 haplotypes) and *SSIIa* (8 haplotypes), indicating that *SSIIIa* had a higher density of functional variants than the other starch synthase genes in the Asian rice population (Figure 5c). The higher functional variant density of *SSIIIa* might arise from spontaneous mutation or human selection.

The expressions of *SSIIIa* in AWS exceeded that of Nipponbare at 25 DPA (Figure 1a). Promoter architecture analysis illustrated the identification of several NAC TF core binding motif and CAAT‐box motif within 2000 bp upstream of the start codon. Among NAC TFs, *OsNAC24* can bind and activate the expression of *SSIIIa* (Jin *et al*., 2023). Among NF‐Y TFs that can bind to CCAAT‐box motif, *NF‐YC12* might regulate *SSIIIa* expression. *SSIIIa* expression was dramatically down‐regulated in the *NF‐YC12* knockout mutant (Xiong *et al*., 2019). *O. meridionalis* displayed the highest expression level of *OsNAC24* among the three genotypes, whereas *O. rufipogon* exhibited no significant difference compared to Nipponbare. Similarly, the expression of *NF‐YC12* in *O. meridionalis* surpassed that of Nipponbare, whereas *NF‐YC12* expression of *O. rufipogon* significantly differed from that of Nipponbare at 25 DPA (Figure S8). Altogether, the interaction between these TFs and other TFs may lead to different expression levels of *SSIIIa* among three genotypes.

## Discussion

### Differential expression of SSRGs between AWS and Nipponbare and its relation to starch physiochemical properties

In this study, we decipher the differences in starch synthesis pathways between Asian cultivated rice, Nipponbare and AWS using RNA‐seq data and examine their relationship to genetic structure using high‐quality genomic data and long‐read Iso‐seq data. Generally, as the seed matured, the majority of SSRGs were downregulated, indicating that starch synthesis occurs most actively during the early stages of seed development. Despite the similar expression of several SSRGs, the expression levels and patterns of most SSRGs varied among the three genotypes. However, several genes, *GBSSI*, *BEIIb*, *SSIIa* and *SSIIIa*, differed in AWS relative to Nipponbare. The other genes, *BEI* and *SSI*, differentiated *O. rufipogon* from *O. meridionalis*. *GBSSI* is one of the essential starch genes responsible for the elongation of the amylose chain. Consequently, the higher expression level and splicing efficiency of *GBSSI* in AWS explain their high amylose content (AC). Tikapunya *et al*. (2018) reported that the Australian population of *O. rufipogon* had an AC ranging from 28% to 32%, whereas *O. meridionalis* displayed a range of 31 to 38%. On the contrary, Nipponbare possessed an AC of 24.5%. Furthermore, previous studies reported that CT repeats ((CT)_n_) in the 5’UTR of *GBSSI* are associated with AC where high amylose rice cultivars were associated with shorter repeat (Biselli *et al*., 2014; Cheng *et al*., 2012; Naseer *et al*., 2021). Nipponbare contains 18 CT repeats, denoted as (CT)_18_, IR64 has (CT)_17_, whereas AWS possesses shorter repeats with (CT)_7_ (Figure S9). *GBSSI* is a major gene that regulates eating and cooking quality (ECQ), as it not only controls amylose content but also influences gel consistency (GC). An SNP C to T in exon 10 of *GBSSI*, resulting in a proline to serine chance, is responsible for gel consistency (GC). In high amylose rice cultivars, those with a C possessed soft GC, whereas those with T exhibited hard GC. Consequently, those with C had a softer texture when freshly cooked than those with T but became much harder after storage for 24 h at 4 °C or showing significant retrogradation (Tran *et al*., 2011). A high AC and C in exon 10 could explain a high retrogradation of AWS as reported by Tikapunya *et al*. (2018).

Amylose fine structure is essential for some functional properties, including digestion rate (Syahariza *et al*., 2013). Increasing the amount of short to medium amylose chains can slow the in vitro digestion rate (Gong *et al*., 2019). Although some *GBSSI* SNPs can influence amylose content, they do not modify amylose chain‐length distribution or amylose fine structure (Wang *et al*., 2015). Previous studies suggested that *BEI* are involved in the synthesis of short amylose chains (Li *et al*., 2015; Nakamura *et al*., 2010; Tappiban *et al*., 2022). *O. meridionalis* exhibited a high expression level compared to Nipponbare and *O. rufipogon* at 25 DPA (Figure 1a). This finding explains why *O. meridionalis* had more amylose short chains, causing a slower in vitro digestion rate compared to some domesticated rice (Zhao *et al*., 2021). We also illustrated that a higher expression of *BEI* may be due to the presence of the strong *OsNAC24* motif in *O. meridionalis*. Zhao *et al*. (2021) also reported that other AWS, that is, *O. australiensis* and *O. officinalis*, had more amylose short chains. To confirm our hypothesis, we also performed promoter structural analysis in *O. australiensis* and *O. officinalis. O. australiensis* and *O. officinalis* had two strong *OsNAC24* motifs similar to that of *O. meridionalis*. Moreover, they also had six weak *OsNAC24* motifs and three bZIP motifs (Figure S10). Shi *et al*. (2023) reported a 6 bp motif called W‐box was responsible for the expression of *OsAUX5*. Two haplotypes divided 215 rice accessions based on the number of W‐box in the promoter. The rice accessions with 2 W‐box motifs had a higher expression of *OsAUX5* than those with 1 W‐box motif. Their study highlighted the importance of CREs for elevating gene expression. *BEI* not only can generate branches in amylose, but it can also form intermediate amylopectin chains, which *SSIIIa* can further elongate to form long amylopectin chains (Nakamura *et al*., 2010). The expression of *BEI* and *SSIIIa* in *O. meridionalis* is higher than that of Nipponbare, which explains the higher amount of long amylopectin chain in *O. meridionalis* compared to Nipponbare (Kasem *et al*., 2014; Tikapunya *et al*., 2017).

The other gene that differentiates AWS from Nipponbare is *SSIIa*. Although the expression levels of *SSIIa* are not significantly different, *SSIIa* in AWS is considered more active due to carrying the *ALK* ^*c*^ allele. *SSIIa* extends short A and B1 chains to generate long B1 chains and modulate gelatinization temperature (GT) (Nakamura *et al*., 2005). The *ALK* ^*c*^ rice cultivars have high GT, while rice cultivars with *ALK* ^*a*^ and *ALK* ^*b*^ possess low GT (Bao *et al*., 2006; Chen *et al*., 2020; Nakamura *et al*., 2005; Song *et al*., 2021; Waters *et al*., 2006). Consistent with *ALK* ^*c*^ role on generating high GT, AWS also demonstrated high GT (Tikapunya *et al*., 2018; Zhao *et al*., 2021). The causal relationship between *SSIIa* and *BEIIb* is strong, as they both contribute to the formation of intermediate amylopectin chains. *BEIIb* is specifically involved in the synthesis of amylopectin by transferring short chains (DP 6–7). The newly transferred chains, then, will be elongated by coordinate actions of *SSI* and *SSIIa* (Nakamura *et al*., 2010). AWS demonstrated more active *BEIIb*, leading to generate more amylopectin branches compared to Nipponbare. We hypothesize that active *SSIIa*, *ALK* ^*c*^, are required to present alongside more active *BEIIb* for generating more intermediate B1 chains. We identified two major haplotypes of *BEIIb*, *BEIIb* ^*j*^ and *BEIIb* ^*i*^, separating japonica and indica rice through a single SNP on exon 4. Given that *O. rufipogon* with higher expression of *BEIIb* corresponds with *BEIIb* ^*i*^, we expect that *BEIIb* ^*i*^ has a higher expression level or enzyme activity than *BEIIb* ^*j*^. To test this hypothesis, a haplotype network analysis was generated by combining SNPs in *BEIIb* and *SSIIa*. Six haplotypes were formed; nonetheless, it can be determined that most of *ALK* ^*c*^ is associated with *BEIIb* ^*i*^, whereas *ALK* ^*a*^ is associated with *BEIIb* ^*j*^. In contrast, *ALK* ^*b*^ is split into *BEIIb* ^*i*^ and *BEIIb* ^*j*^ (Figure S11). This finding is also supported by the fact that under the same active *BEIIb*, rice cultivars with *ALK* ^*c*^ generated more B1 chains and lower A chains than those with *ALK* ^*b*^, whereas under inactive *BEIIb*, rice cultivars with *ALK* ^*c*^ failed to create more B1 chains compared to those with *ALK* ^*b*^ (Hu *et al*., 2023). This finding also explains that a higher proportion of B1 chains in *O. rufipogon* and *O. meridionalis* compared to Nipponbare (Kasem *et al*., 2014; Tikapunya *et al*., 2017) is due to higher expression of *BEIIb* and more active *SSIIa*.

### How domestication modified starch synthesis pathways for better eating and cooking qualities

The origin of Asian rice is in debate, whether from a single or multiple ancestors. For example, a recent study suggested that Asian rice was initially domesticated as a single crop, which is the hybrid offspring of *O. rufipogon* and *O. nivara*, which then diverged into two major subgroups, japonica and indica, through human selection (Lu *et al*., 2022). However, analysis of the chloroplast genome variation in domesticated rice strongly supports two maternal domestications (Moner *et al*., 2020). Crop domestication involves not only its origin but also the selection of preferred traits. Here, we explain how rice domestication has modified starch synthesis pathways for better eating and cooking quality. Three physicochemical properties: AC, GC and GT influenced ECQ (Tian *et al*., 2009). Previous studies indicated that rice cultivars with low‐to‐intermediate AC generally exhibit better palatability, stickiness and hardness compared to high‐AC rice cultivars (Huang *et al*. 2020; Tian *et al*., 2009; Zhang *et al*., 2019; Zhou *et al*., 2021).

**Figure 6:**
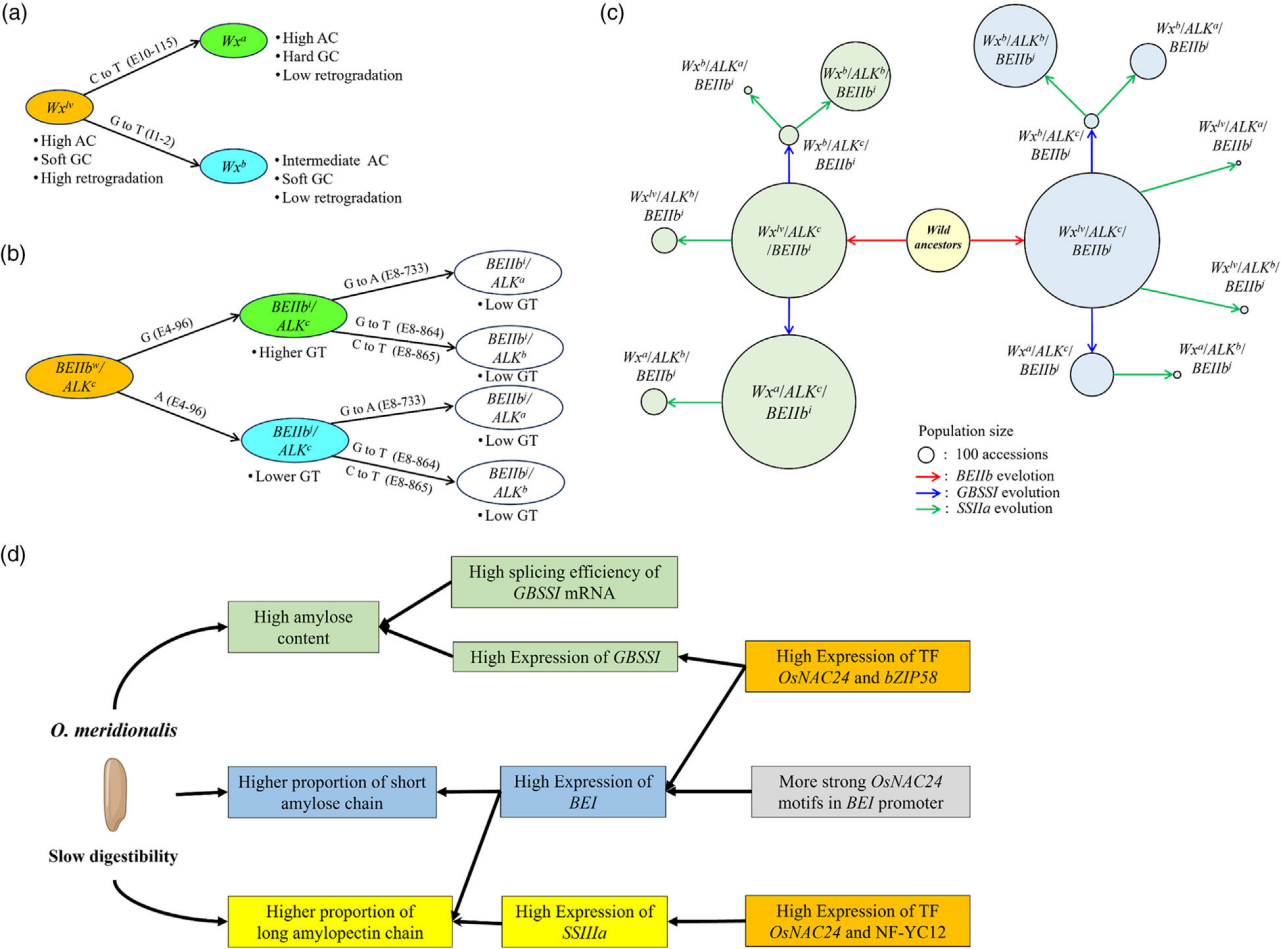
Evolution of key starch synthesis genes and their relationship with starch physiochemical properties. (a) *GBSSI* evolution and its relationship with starch physiochemical properties. (b) Proposed evolutionary pathway of combined *BEIIb* and *SSIIa* haplotypes and its relation to GT. (c) Proposed evolutionary relationship among various combined haplotypes of *BEIIb, GBSSI* and *SSIIa* in Asian rice accessions. The background is colour‐coded, with green representing the *BEIIb* ^*i*^ haplotype and blue representing the *BEIIb* ^*j*^ haplotype. (d) Proposed model illustrating the roles of specific starch‐related genes and their cis‐regulatory elements (CREs) in modulating starch digestibility rates.

Two important SNPs in *GBSSI*, G to T in intron 1 (I1‐2) and C to T in exon 10 (E10‐115) differentiate Asian rice ancestors (*O. rufipogon* and *O. nivara*) from *Wx* ^*a*^ and *Wx* ^*b*^. Both ancestors exhibit the G nucleotide in I1‐2 and C in E10‐115, suggesting that the G nucleotide in I1‐2 and C in E10‐115 is ancestral and plays a significant role in rice domestication. Consequently, the changes in those positions may result from mutations that were further selected by humans. The G to T in I1‐2 decreases the levels of mature mRNA (Cai *et al*., 1998; Hirano *et al*., 1998), while C to T in exon 10 (E10‐115) reduces *GBSSI* enzyme activity to a moderate level by increasing phosphorylation level (Zhang *et al*., 2019). Furthermore, C to T in E10‐115 also affected GC and retrogradation levels (Tran *et al*., 2011; Zhang *et al*., 2019). Rice ancestors (*Wx* ^*lv*^) possess high AC and soft GC, resulting in poor eating quality (Zhang *et al*., 2019) and high retrogradation (Tran *et al*., 2011). Two separate *GBSSI* evolutions improve the eating quality of Asian domesticated rice. *Wx* ^*b*^ which is commonly found in japonica rice improves eating quality by reducing AC as a result of G to T mutation in intron 1. In contrast, *Wx* ^*a*^, often present in indica rice, demonstrates T in E10‐155. This mutation makes AC in *Wx* ^*a*^ cultivars not as high as *Wx* ^*lv*^ (Feng *et al*., 2021). Moreover, although *Wx* ^*a*^ cultivars have higher AC, which results in a firmer texture compared to *Wx* ^*b*^ cultivars, their hard GC leads to low retrogradation, meaning the hardness does not change significantly after cool storage. In contrast, the soft GC of *Wx* ^*lv*^ results in high retrogradation, leading to greater hardness after storage (Figure 6a). Regarding the two SNPs, AWS (*O. meridionalis* and Australian populations of *O. rufipogon*) possess similarities with *Wx* ^*lv*^. However, AWS contain two unique functional SNPs that need further investigation.

GT is responsible for rice cooking quality. During domestication, rice cultivars with low GT might have been selected for their cooking quality by humans (Waters *et al*., 2006). *SSIIa* is a major gene controlling GT. However, some additive genes are also responsible for GT, including *BEIIb* (Tian *et al*., 2009). Both Asian wild ancestors possess *ALK* ^*c*^, an active *SSIIa* allele, while two major *BEIIb* haplotypes, *BEIIb* ^*j*^ and *BEIIb* ^*i*^, are identified among Asian domesticated rice derived from *O. rufipogon* and *O. nivara*, respectively. Because the two ancestors possess similar *ALK* ^*c*^ alleles but different *BEIIb* alleles, two independent evolutions might have occurred: *BEIIb* ^*i*^/*ALK* ^*c*^ and *BEIIb* ^*j*^/*ALK* ^*c*^. Mutations in *ALK* ^*c*^ can result in six possible allele combinations: *BEIIb* ^*i*^/*ALK* ^*c*^, *BEIIb* ^*i*^/*ALK* ^*b*^, *BEIIb* ^*i*^/*ALK* ^*a*^, *BEIIb* ^*j*^/*ALK* ^*c*^, *BEIIb* ^*j*^/*ALK* ^*b*^ and *BEIIb* ^*j*^/*ALK* ^*a*^ (Figure 6b). Altogether, *GBSSI*, *BEIIb* and *SSIIa* are responsible for ECQ, which have been target genes for better ECQ during domestication. A schematic evolution and distribution in the Asian rice population of the three gene haplotypes was performed (Figure 6c; Table S12). A total of 15 allele combinations are separated into two groups based on *BEIIb* backgrounds. Regardless of *BEIIb* backgrounds, the ancestral allele combination (*Wx* ^*lv*^/*ALK* ^*c*^) is frequently found in Asian rice‐cultivated populations. In the *BEIIb* ^*i*^ background, *Wx* ^*a*^/*ALK* ^*c*^ constitutes the predominant allele combination. Whereas, in the *BEIIb* ^*j*^ background, *Wx* ^*b*^/*ALK* ^*b*^ is the second most often identified after ancestral alleles *Wx* ^*lv*^/*ALK* ^*c*^. These findings suggest that mutations and human selection have improved ECQ. Furthermore, rice starch gene analysis, especially in *BEIIb*, indicated that the ancestors of Asian domesticated rice are two wild species, *O. rufipogon* and *O. nivara*.

In this study, we also found that one of the differences between AWS is the expression of *BEI*. *O. meridionalis* had a higher *BEI* expression than that of *O. rufipogon*, which might explain a higher amount of short‐medium amylose chains in *O. meridionalis*. Gong *et al*. (2019) indicated that amylose fine molecular structures play a crucial role in influencing the digestion of retrograded starch. During retrogradation, double helices are formed between amylose molecules. Rice with a higher amount of amylose short‐medium chains formed faster and stronger interactions, resulting in tightly packed small and thin cells. Densely packed small cells inhibit their susceptibility to digestive enzymes, leading to a slower digestion rate. However, a faster recrystallization rate during the short‐term retrogradation process resulted in a harder texture of cooked rice (Li et al., 2021). Therefore, *O. meridionalis* might have poorer eating quality than that of Asian wild ancestors. Consequently, poor ECQ may have prevented humans from selecting *O. meridionalis* during rice domestication.

### Desired starch gene haplotypes for the development of slowly digested starch cultivars

Three starch characteristics have been identified as factors contributing to slower starch digestibility in *O. meridionalis*, that is, high AC, a higher proportion of short amylose chains and a higher proportion of long amylopectin chains. A higher AC reduces the digestibility of starch due to a positive correlation with the formation of RS (Sajilata *et al*., 2006; Raigond *et al*., 2015; Shen *et al*., 2022). However, several studies reported some rice varieties with relatively similar high AC differ in starch digestibility due to variations in amylose molecular fine structure (Panlasigui *et al*., 1991; Zhao *et al*., 2021). A higher proportion of short amylose chains leads to a slower digestion rate (Gong *et al*., 2019; Syahariza *et al*., 2013; Zhao *et al*., 2021). Similarly, the molecular fine structure of amylopectin, particularly long amylopectin chains, contributes to a slower digestion rate (Li *et al*., 2021).

Based on the findings described in this study, we propose a model for the role of several genes and their CREs in reducing starch digestibility rate (Figure 6d). Two main factors affecting high AC in *O. meridionalis* are the high splicing efficiency of *GBSSI* mRNA and high expression of *GBSSI*. A higher expression of *BEI* is responsible for both amylose and amylopectin chain length distributions. The higher expression of *BEI* in *O. meridionalis* is due to the higher expression of two TFs, *OsNAC24* and *OsbZIP58*, as well as more strong *OsNAC24* motifs in the promoter. Likewise, the higher expression of *SSIIIa* in *O. meridionalis* is due to the higher expression of two TFs, *OsNAC24* and *NF‐YC12*. Altogether, *GBSSI*, *BEI* and *SSIIIa* haplotypes in *O. meridionalis* might be utilized to develop slowly digested rice cultivars.

The introgression of starch‐related genes from AWS, particularly *O. meridionalis*, offers significant potential for enhancing carbohydrate quality in domesticated rice. Specifically, the incorporation of more active alleles of *GBSSI*, *BEI* and *SSIIIa* from *O. meridionalis* may result in slower starch digestibility, a trait associated with high carbohydrate quality. Both conventional and modern breeding approaches can be utilized for this purpose. While the transfer of traits from wild to cultivated rice through hybridization often encounters challenges such as fertilization barriers and hybrid sterility, progress has been made in overcoming these obstacles. For instance, chromosome‐segment substitution lines have been successfully developed between *O. meridionalis* and cultivated rice (Arbelaez *et al*., 2015; Yoshimura *et al*., 2010), demonstrating the feasibility of introgressing beneficial genes. However, linkage drag that might carry domestication‐related traits, such as seed dormancy, seed shattering and photoperiod sensitivity, as well as inferior yield‐related traits, might delay the progress in incorporating these traits into domesticated rice. Overcoming linkage drag is a critical challenge and requires precise genetic tools and approaches. Recent advances in genome editing technologies using the CRISPR/Cas9 system, such as base and prime editing, have enabled precise modification of SNPs and small indels within the genes, enabling the introgression of beneficial traits without the co‐transfer of undesirable characteristics. The comprehensive comparative genomics and transcriptomics of key starch‐related genes presented in this study provide a valuable foundation for modifying starch characteristics through CRISPR/Cas9 technologies. Furthermore, the findings not only provide insight into the evolution of starch gene synthesis during domestication but also pave the way for unlocking desirable gene haplotypes of wild rice for developing slowly digested starch cultivars.

## Materials and methods

### Selection of starch‐synthesis‐related genes

A total of 72 SSRGs were selected from a literature search (Table S1). The 72 genes represent five classes of starch biosynthesis enzymes (AGPase, SS, SBE, DBE and Pho), transcription factors and other related genes.

### RNA‐seq analysis

RNA‐seq data (PRJNA819483) was downloaded from the Sequence Read Archive (SRA) at NCBI (http://www.ncbi.nlm.nih.gov/sra/). The data consists of three stages (5‐, 15‐ and 25‐days post anthesis (DPA)), representing early, middle and late stages of seed development of Nipponbare and two AWS (*Oryza rufipogon* and *Oryza meridionalis*) in three replications (Hasan *et al*., 2022a). The RNA‐seq data were analysed using Galaxy (www.usegalaxy.org.au). High‐quality reads were first aligned to the rice reference genome using HISAT2. The results were subjected to feature counts to obtain the read counts of all samples. The read counts of the genes were used to measure gene expression based on the reads per kilobase of transcript per million reads mapped (RPKM). The differentially expressed genes (DEGs) were used to compare Nipponbare and AWS in three different stages of seed development. DEGs were identified using DESeq2 (*P*‐adj <0.01).

### Variant analysis

The 72 gene sequences of the reference plant, Nipponbare, were retrieved from the Rice Annotation Project Database (https://rapdb.dna.affrc.go.jp/index.html) and the transcript IDs of the genes are shown in Table S2. The exon‐intron structural features of the genes were annotated using Geneious Prime V2023.0.4. The genes were mapped to the *O. rufipogon* Australia population and *O. meridionalis* genomes using Geneious Mapper with the highest sensitivity. The sequences relative to the original reference gene sequences, or orthologous genes, were extracted. The orthologous genes of each wild species were subsequently aligned to the reference genes using Geneious pairwise alignment. The global alignment was conducted with free end gaps and a cost matrix of 93% similarity. The alignments were then employed for SNP and indel identification. The coding DNA sequence (CDS) of each gene was translated into amino acids and utilized to determine synonymous and nonsynonymous amino acid changes between Nipponbare and AWS.

### Phylogenetic analysis of wild and domesticated rice

To understand starch gene relationships in wild and domesticated rice species, a phylogenetic tree was constructed for 19 key starch genes. The 19 genes were three plastidic translocator genes (*GPT1*, *BT1* and *PGlcT*), four AGPase genes (*AGPS1, AGPS2, AGPL1 and AGPL2*), four starch synthase (SS) genes (*GBSSI, SSI*, *SSIIa* and *SSIIIa*), two branching enzyme (BE) genes (*BEI* and *BEIIb*), two debranching enzyme (DBE) genes (*ISA1* and *ISA2*), two phosphorylases (*PHO1* and *PHO2*) and two plastidial disproportionating enzyme genes (*DPE1* and *DPE2*). The wild and cultivated rice varieties studied included Asian varieties (Nipponbare, Kitaake, IR8 and N22) and their ancestors (*O. rufipogon* and *O. nivara*), African rice (*O. glaberrima* and *O. barthii*) and Australian wild rice species (*O. rufipogon* Australian population and *O. meridionalis*). The concatenated genes of 10 rice species were subjected to multiple sequence alignment using the MAFFT alignment tool in Geneious Prime V2023.0.4 with default parameters. The phylogenetic tree was constructed using Maximum likelihood (ML) and Bayesian inference (BI) methods. The ML was performed using the Randomized Axelerated Maximum Likelihood (RAxML) method employing a generalized time reversible (GTR) GAMMA nucleotide substitutional model with 1000 bootstrap replicates (Silvestro and Michalak, 2012). The BI analysis was performed using MrBayesversion 3.2 (Ronquist *et al*., 2012) with GTR substitution model, gamma rate variation and other default parameters in Geneious Prime V2023.0.4. Leersia perrieri was used as the outgroup species. The final phylogenetic tree was further modified in iTOL version 6.5.2 (Letunic and Bork, 2019).

### Haplotype analysis

Haplotype analysis was performed by comparing AWS with 4726 Asian rice (*O. sativa*) accessions. The SNP and Indel (variants) data for 4726 Asian rice accessions were available at RiceVarMap (http://ricevarmap.ncpgr.cn/), accessed in August 2024. The variant data was obtained from the gene sequences. The variants were further analysed for the position within the gene. Subsequently, SNPs observed in exons were classified as nonsynonymous/functional SNPs, which result in the substitution of amino acid residues, or synonymous SNPs, which do not change the amino acid encoded. The nonsynonymous SNPs were then used to construct a haplotype network generated on the RiceVarMap website.

### Iso‐seq analysis

The Iso‐seq data was prepared from *O. rufipogon and O. meridionalis*. The details of Iso‐seq methods are available in Supplementary methods. Nipponbare Iso‐seq data (PRJNA813759) was downloaded from the Sequence Read Archive (SRA) at NCBI (http://www.ncbi.nlm.nih.gov/sra/). The Iso‐seq data were analysed according to Hasan *et al*. (2022b), with some modifications. The total transcript, including high‐quality and low‐quality transcripts, was subjected to CD‐Hit analysis with a 99% sequence identity threshold to eliminate isoform redundancy using OmicsBox software (v.2.0). The SQANTI3 analysis was then conducted to map the final isoforms post CD‐Hit.

## Funding information

This research was supported by the ARC Centre of Excellence for Plant Success in Nature and Agriculture (CE200100015).

## Author contributions

Study conception and design: Henry RJ, Nurmansyah; data collection: Nurmansyah, Furtado A; analysis and interpretation of results: Nurmansyah, Henry R, Furtado A, Okemo P; draft manuscript preparation: Nurmansyah. All authors reviewed the results and approved the final version of the manuscript.

## Conflict of interest

The authors declare that they have no conflict of interest.

## Supporting information

Supplementary methods.
**Figure S1** Hierarchical clustering of the expression level of 72 SSRGs at three stages of seed development. Each row represents a gene, and each column represents a genotype within the seed development stages. The expression level indicated by the colour grids is based on log_2_ (RPKM+1), green and red colour representing high and low expression levels, respectively.
**Figure S2** Analysis of DEGs between 15 DPA/5 DPA, and 25 DPA/15 DPA in three genotypes. (a) Total number and distribution of DEGs in 15 DPA/5 DPA. (b) Total number and distribution of DEGs in 25 DPA/15 DPA. (c) Venn diagram showing similar DEGs between genotypes in 15 DPA/5 DPA. (d) Venn diagram showing similar DEGs between genotypes in 25 DPA/15 DPA.
**Figure S3** Analysis of DEGs among genotypes at three developing stages. (a) Total number and distribution of DEGs in 72 SSRGs between *O. rufipogon* and Nipponbare. (b) Venn diagram showing similar DEGs between developing stages between *O. rufipogon* and Nipponbare. (c) Fold change value of DEGs (*P‐adj* < 0.01) between *O. rufipogon* and Nipponbare. (d) Total number and distribution of DEGs in 72 SSRGs between *O. meridionalis* and Nipponbare. (e) Venn diagram showing similar DEGs between developing stages between *O. meridionalis* and Nipponbare. (f) Fold change value of DEGs (*P‐adj* < 0.01) between *O. meridionalis* and Nipponbare.
**Figure S4** Phylogenetic tree of 72 genes of Nipponbare and AWS. (a) Phylogenetic tree based on CDS sequences. (b) Phylogenetic tree based on protein sequences.
**Figure S5** Haplotype network of *BEI* based on Asian rice accessions.
**Figure S6** SSI promotor analysis. (a) ACC element comparison between AWS and Nipponbare. (b) The expression of *OsNAC20* and *OsNAC26* in three seed development stages across three genotypes. (c) SNP and Indel resulted in different CRE motifs between AWS and Nipponbare. (d) The expression of *NY‐YA8* in three seed development stages across three genotypes.
**Figure S7** *ALK* allele distribution in Asian rice cultivars.
**Figure S8** The expression of *OsNAC24* and *NF‐YC12* in three seed development stages across three genotypes.
**Figure S9** CT repeats differences in *GBSSI* among AWS, Nipponbare, and IR64.
**Figure S10** Promoter architecture of *BEI* among Nipponbare, *O. meridionalis*, *O. australiensis*, and *O. officinalis*. Green annotation indicates similar motifs, including TATA box and MADS box. Red and blue annotations indicate strong and weak *OsNAC24* motifs, respectively.
**Figure S11** Combined haplotypes of *BEIIb* and *SSIIa* and their distribution in Asian rice accessions.
**Table S1** Selected starch‐synthesis‐related genes.
**Table S2** Transcript identity of 72 starch‐synthesis‐related genes.
**Table S3** Gene classification based on expression level.
**Table S4** 123 variants of *GBSSI* identified in 4,726 rice accessions.
**Table S5** Variations in eight *GBSSI* haplotypes identified in selected rice accessions.
**Table S6** Classification of AS events.
**Table S7** Six SNPs observed in *BEI* between Nipponbare and *O. meridionalis*.
**Table S8** Haplotype classification of *BEI* in Asian rice accessions.
**Table S9** RPKM values of putative transcription factor genes acting on *BEI*.
**Table S10** Haplotype classification of *BEIIb* in Asian rice accessions.
**Table S11** Haplotype classification of *SSIIa*.
**Table S12** Combined haplotypes of *BEIIb, GBSSI*, and *SSIIa* in Asian rice accessions.
Supplementary References.

## Data Availability

The raw data of RNA‐seq and Nipponbare Iso‐seq can be downloaded from the Sequence Read Archive (SRA) at NCBI (http://www.ncbi.nlm.nih.gov/sra/) under the BioProject number PRJNA819483 and PRJNA813759, respectively. The rice reference genome and annotation of *O. sativa* var. Nipponbare are available in the Rice Annotation Project Database (https://rapdb.dna.affrc.go.jp/) and the Rice Genome Annotation Project (http://rice.uga.edu/). The wild and domesticated rice cultivars (Asian *O. rufipogon, O. nivara, O. barthii, O. glaberrima*, IR64 and N22) can be downloaded from Ensembl Plants (https://plants.ensembl.org/index.html).
