# Supplementary material for: Unique starch biosynthesis pathways in wild rice revealed by multi‐omics analyses
Source: Plant Biotechnol J. 2025 Mar 27;23(6):2429–45. doi: 10.1111/pbi.70021 (PMC12120894; doi:10.1111/pbi.70021)
Supplement: Supplementary file 1 — Supplementary methods. Figure S1 Hierarchical clustering of the expression level of 72 SSRGs at three stages of seed development. Each row represents a gene, and each column represents a genotype within the seed development stages. The expression level indicated by the colour grids is based on log2 (RPKM+1), green and red colour representing high and low expression levels, respectively. Figure S2 Analysis of DEGs between 15 DPA/5 DPA, and 25 DPA/15 DPA in three genotypes. (a) Total number and distribution of DEGs in 15 DPA/5 DPA. (b) Total number and distribution of DEGs in 25 DPA/15 DPA. (c) Venn diagram showing similar DEGs between genotypes in 15 DPA/5 DPA. (d) Venn diagram showing similar DEGs between genotypes in 25 DPA/15 DPA. Figure S3 Analysis of DEGs among genotypes at three developing stages. (a) Total number and distribution of DEGs in 72 SSRGs between O. rufipogon and Nipponbare. (b) Venn diagram showing similar DEGs between developing stages between O. rufipogon and Nipponbare. (c) Fold change value of DEGs (P‐adj < 0.01) between O. rufipogon and Nipponbare. (d) Total number and distribution of DEGs in 72 SSRGs between O. meridionalis and Nipponbare. (e) Venn diagram showing similar DEGs between developing stages between O. meridionalis and Nipponbare. (f) Fold change value of DEGs (P‐adj < 0.01) between O. meridionalis and Nipponbare. Figure S4 Phylogenetic tree of 72 genes of Nipponbare and AWS. (a) Phylogenetic tree based on CDS sequences. (b) Phylogenetic tree based on protein sequences. Figure S5 Haplotype network of BEI based on Asian rice accessions. Figure S6 SSI promotor analysis. (a) ACC element comparison between AWS and Nipponbare. (b) The expression of OsNAC20 and OsNAC26 in three seed development stages across three genotypes. (c) SNP and Indel resulted in different CRE motifs between AWS and Nipponbare. (d) The expression of NY‐YA8 in three seed development stages across three genotypes. Figure S7 ALK allele distribution in Asian rice cul [file PBI-23-2429-s001.docx]

**Supplementary Information**

**Unique starch biosynthesis pathways in wild rice revealed by multi-omics analyses**

Nurmansyah^1,2,3^, Agnelo Furtado^1,2^¸ Pauline Okemo^1,2^, Robert J. Henry^1,2*^

^1^Queensland Alliance for Agriculture and Food Innovation, University of Queensland, Brisbane, QLD, 4067, Australia

^2^ARC Centre for Plant Success in Nature and Agriculture, University of Queensland, Brisbane, QLD, 4067, Australia

^3^Department of Agronomy, Faculty of Agriculture, Universitas Gadjah Mada, Yogyakarta, Indonesia

*Correspondence: Robert J. Henry [robert.henry@uq.edu.au](mailto:robert.henry@uq.edu.au)

**Supplementary methods**

**Iso-seq sample preparation**

Leaf tissues and seeds from four different development stages (5, 10, 15, and 25 DPA) of *O. rufipogon* and *O. meridionalis* were snap-frozen with liquid nitrogen immediately after collection. Total RNA was extracted separately using the RNA extraction (Furtado, 2014) and purified using the Qiagen RNeasy Mini Kit (Qiagen, Valencia, CA, United States). Equal mass of the total RNA samples was pooled. Aliquots of 900 ng input were prepared for the Iso-Seq cDNA library, as per the procedures for ‘Iso-Seq template preparation for Sequel Systems with size selection’ (PacBio, PN. 101-070-200, version 6, September 2018).

**Iso-seq analysis**

The raw data from the PacBio Iso-sequencing was analyzed using the IsoSeq3 analysis application in SMRT Link V. 6.0 with default parameters. Circular consensus sequences (CCS) were generated using consensus sequences of a minimum of two full-pass subreads in a single zero-mode waveguide (ZMW). The full-length (FL), or non-full-length (NFL) reads were classified based on the presence of the 5′ primer and 3′ primer or 3′ terminal poly-A tail. Reads containing 5′ and 3′ cDNA primers, and a poly-A tail were considered full-length non-chimeric (FLNC) reads, whereas those that lack any of these tags were classified as non-full-length (NFL) reads. The FLNC reads were then trimmed to remove barcoded, unbarcoded cDNA primers and unwanted primers, and turn the reads to the 5 → 3 orientation. We further refined FLNC reads by trimming poly-A tails and removing artificial concatemers. High-quality (HQ) and low-quality (LQ) transcripts of FLNC were classified based on the accuracy percentage of ≥ 99% and < 99%, respectively. The total transcript, including HQ and LQ transcripts, was subjected to CD-Hit analysis with a 99% sequence identity threshold to eliminate isoform redundancy using OmicsBox software (v.2.0).

**Transcriptome Characterization**

The SQANTI3 (Structural and Quality Annotation of Novel Transcripts Isoforms) version 4.2 pipeline was performed to map the final isoforms (Tardaguila et al., 2018). The total transcript of Nipponbare, *O. rufipogon,* and *O. meridionalis* was mapped with minimap2 utilizing their respective reference genomes. An improved Nipponbare reference genome (Abdullah et al., 2024) was used to map the total transcript of Nipponbare. The SQANTI3 output was employed to identify different spliced isoforms of the genes and subsequently validated in Geneious Prime V2023.0.4.


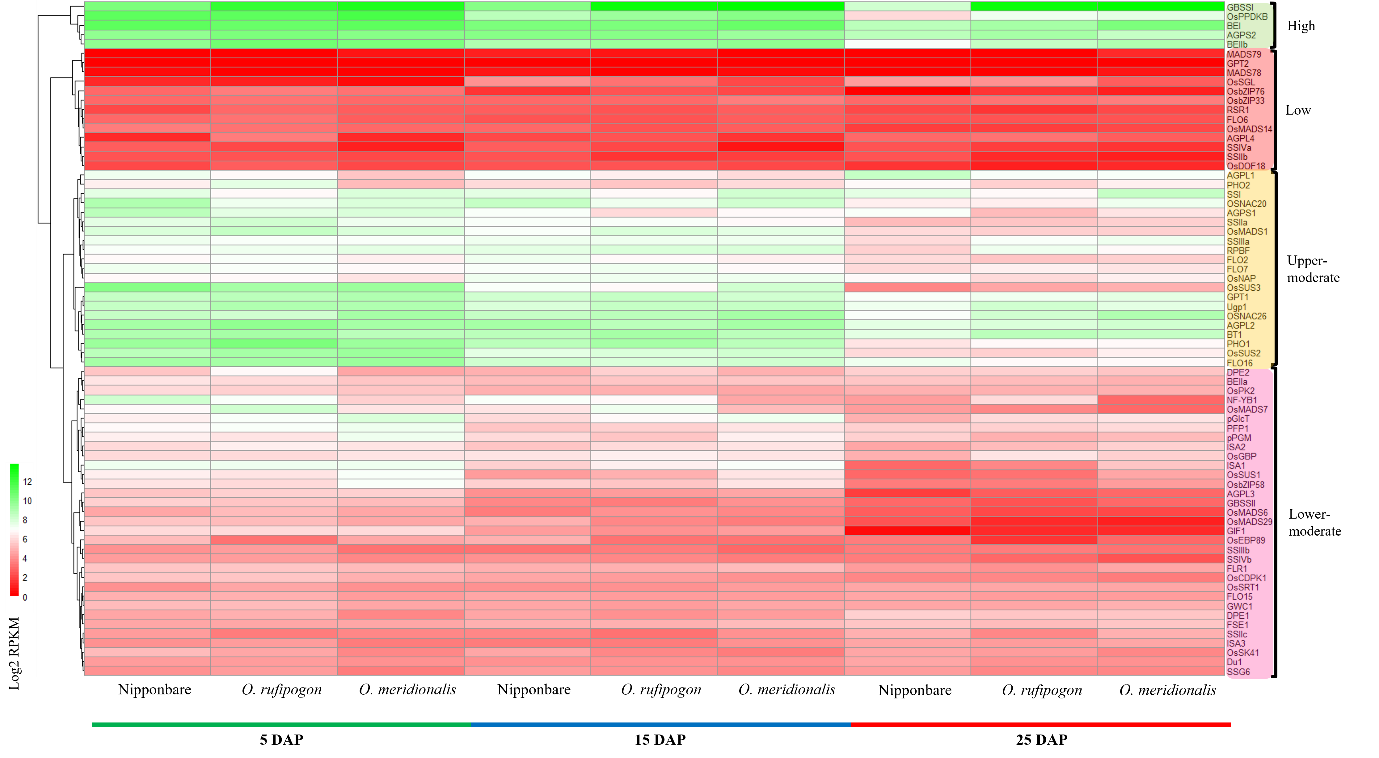


**Fig. S1** Hierarchical clustering of the expression level of 72 SSRGs at three stages of seed development. Each row represents a gene, and each column represents a genotype within the seed development stages. The expression level indicated by the colour grids is based on log_2_ (RPKM+1), green and red colour representing high and low expression levels, respectively.


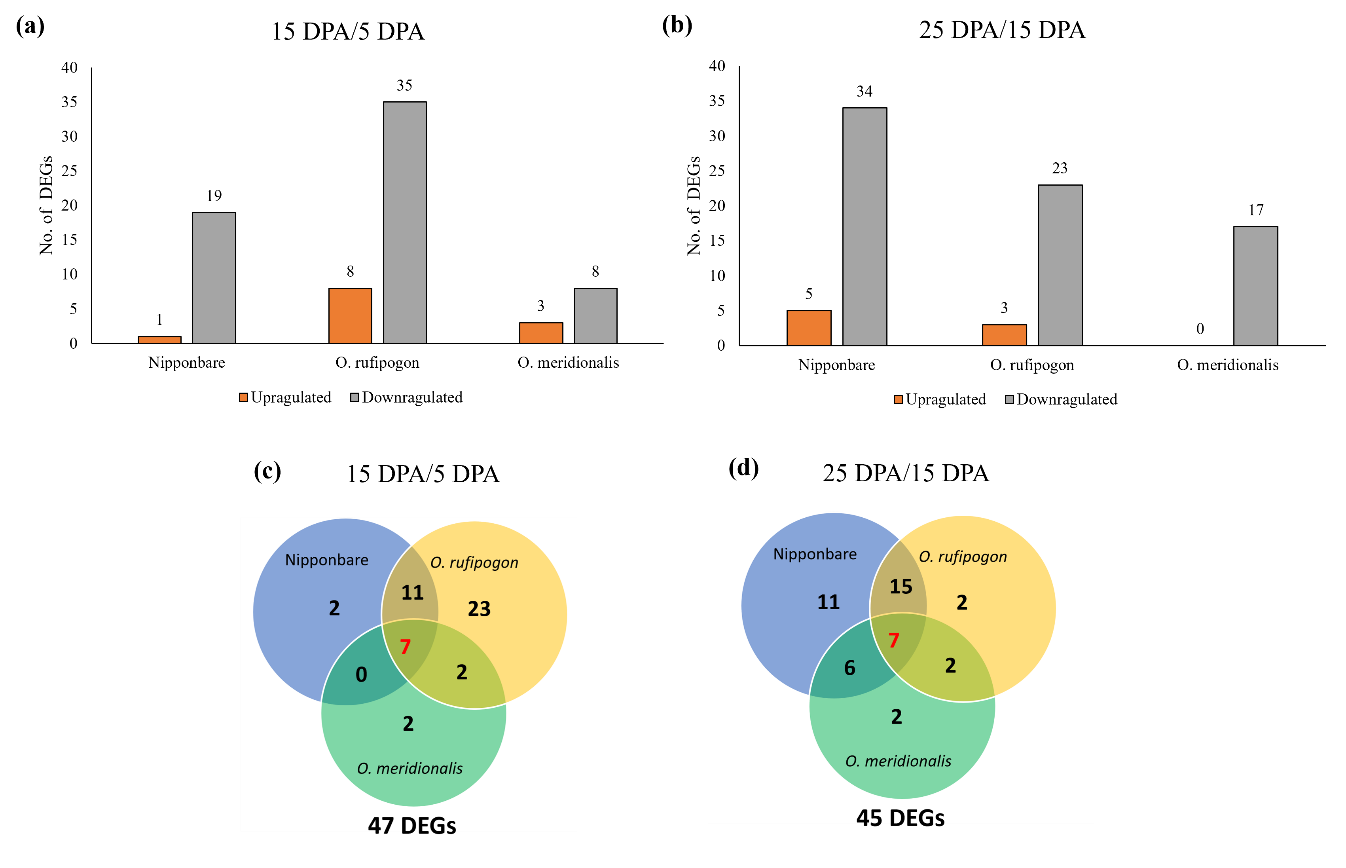


**Fig. S2** Analysis of DEGs between 15 DPA/5 DPA, and 25 DPA/15 DPA in three genotypes. **(a)** Total number and distribution of DEGs in 15 DPA/5 DPA. **(b)** Total number and distribution of DEGs in 25 DPA/15 DPA. **(c)** Venn diagram showing similar DEGs between genotypes in 15 DPA/5 DPA. **(d)** Venn diagram showing similar DEGs between genotypes in 25 DPA/15 DPA.


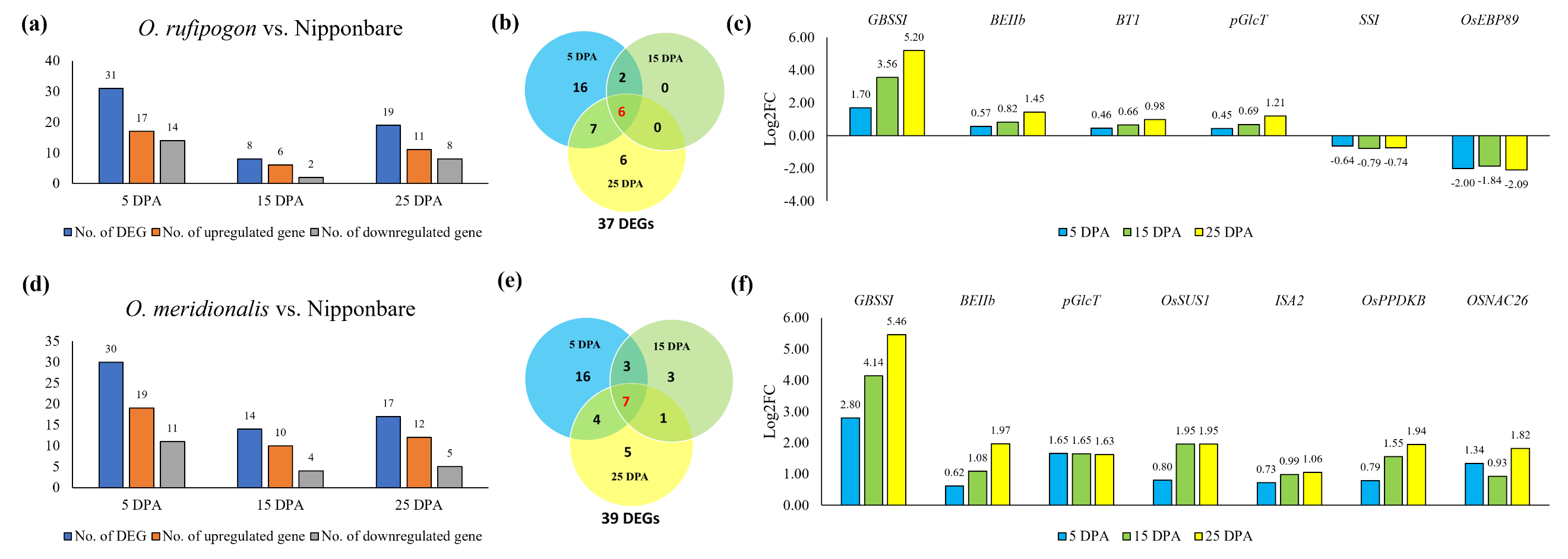


**Fig. S3** Analysis of DEGs among genotypes at three developing stages. **(a)** Total number and distribution of DEGs in 72 SSRGs between *O. rufipogon* and Nipponbare. **(b)** Venn diagram showing similar DEGs between developing stages between *O. rufipogon* and Nipponbare. **(c)** Fold change value of DEGs (*p-adj* < 0.01) between *O. rufipogon* and Nipponbare. **(d)** Total number and distribution of DEGs in 72 SSRGs between *O. meridionalis* and Nipponbare. **(e)** Venn diagram showing similar DEGs between developing stages between *O. meridionalis* and Nipponbare. **(f)** Fold change value of DEGs (*p-adj* < 0.01) between *O. meridionalis* and Nipponbare.


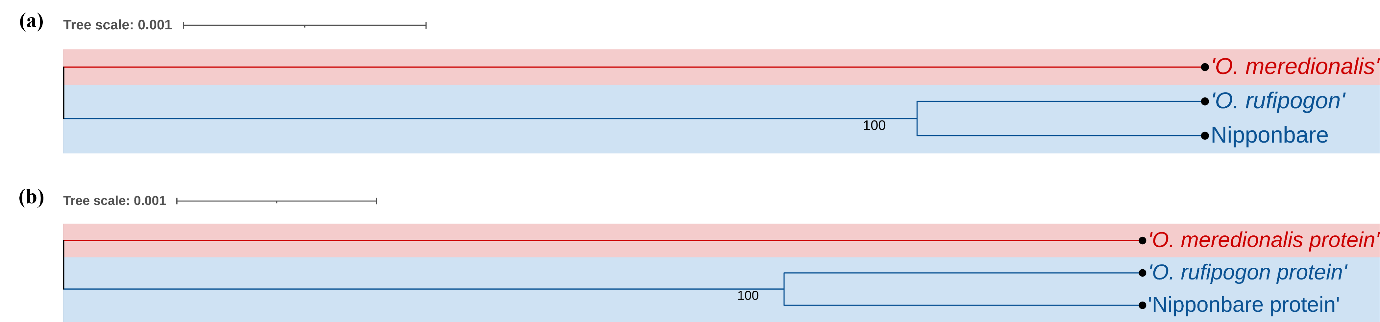


**Fig. S4** Phylogenetic tree of 72 genes of Nipponbare and AWS. **(a)** Phylogenetic tree based on CDS sequences. **(b)** Phylogenetic tree based on protein sequences


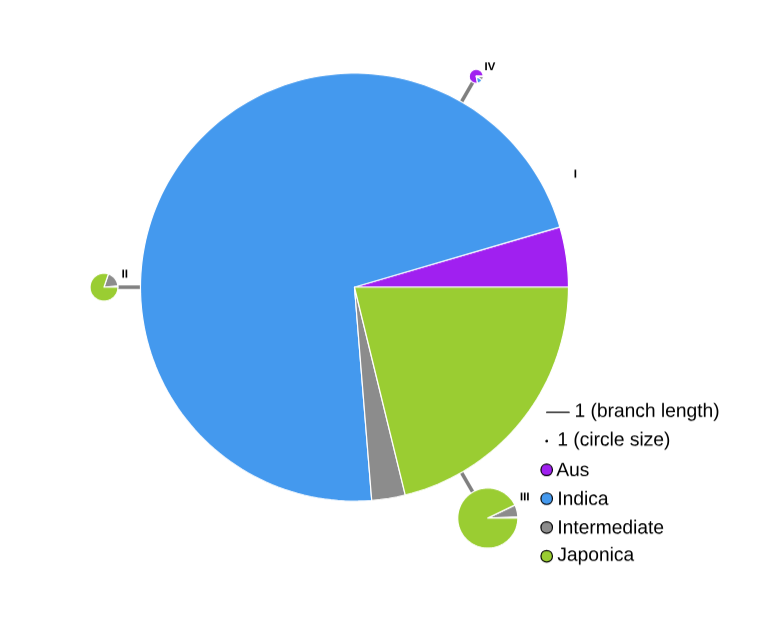


**Fig. S5** Haplotype network of *BEI* based on Asian rice accessions


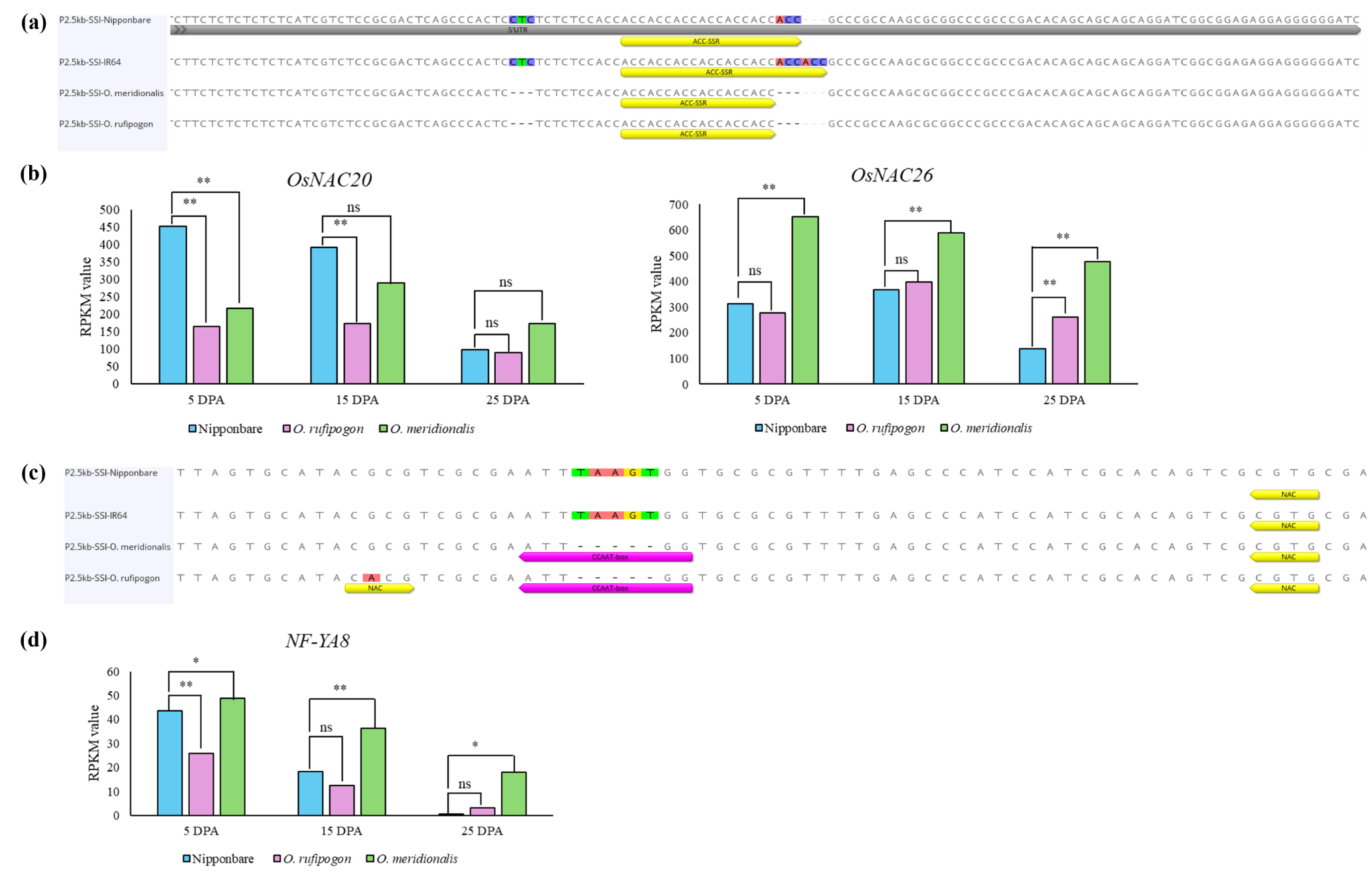


**Fig. S6** SSI promotor analysis. **(a)** ACC element comparison between AWS and Nipponbare. **(b)** The expression of *OsNAC20* and *OsNAC26* in three seed development stages across three genotypes. **(c)** SNP and Indel resulted in different CRE motifs between AWS and Nipponbare. **(d)** The expression of *NY-YA8* in three seed development stages across three genotypes.


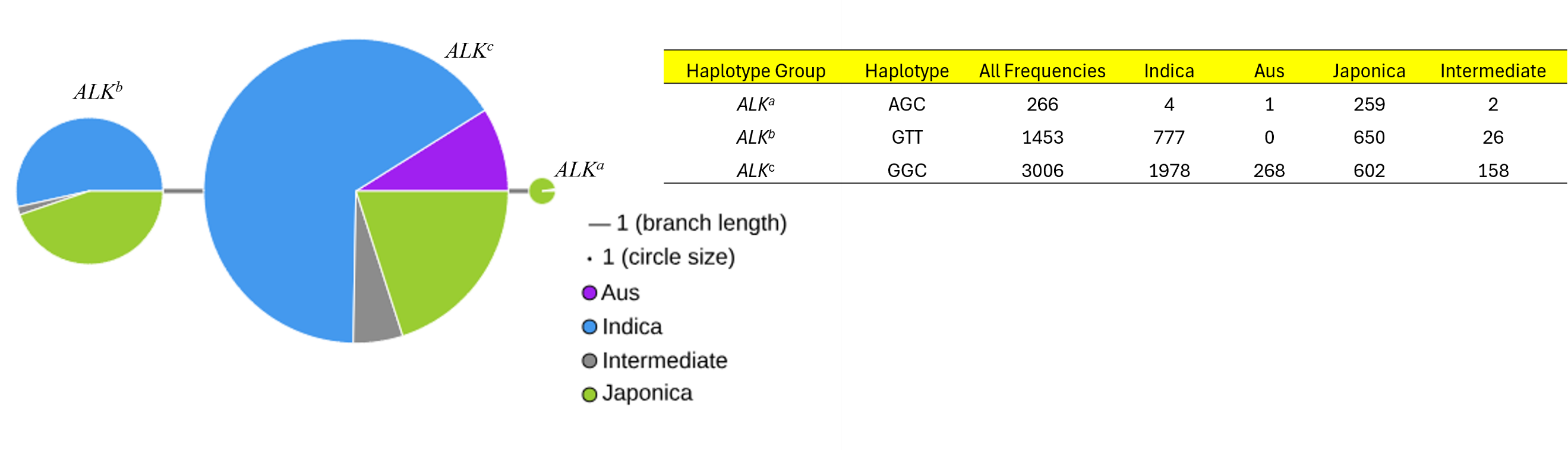


**Fig. S7** *ALK* allele distribution in Asian rice cultivars


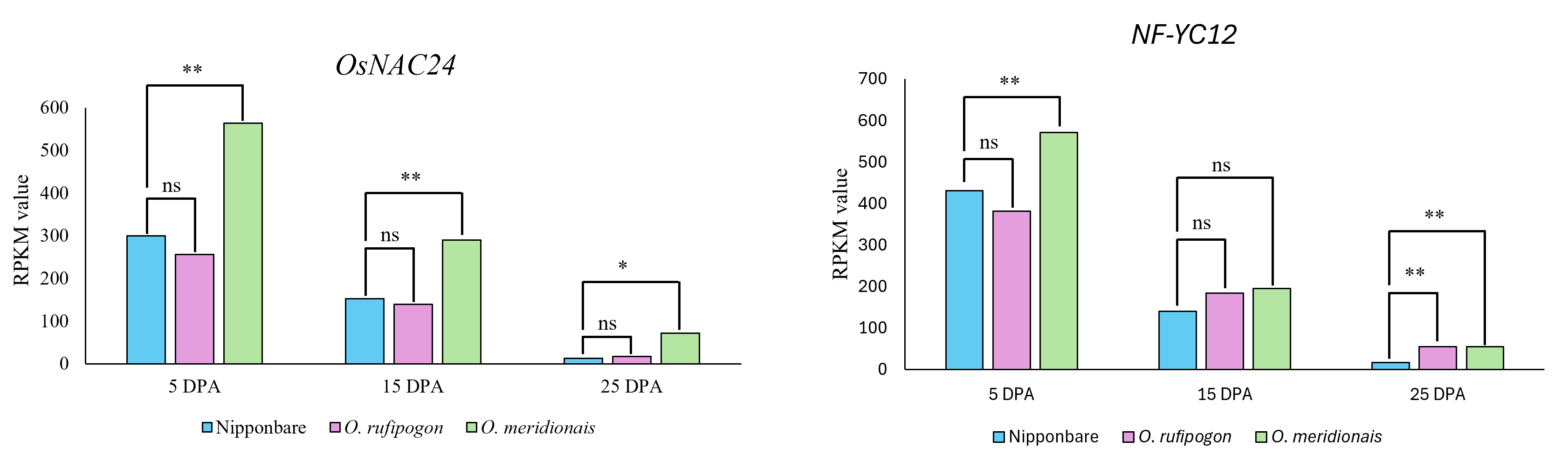


**Fig. S8** The expression of *OsNAC24* and *NF-YC12* in three seed development stages across three genotypes.


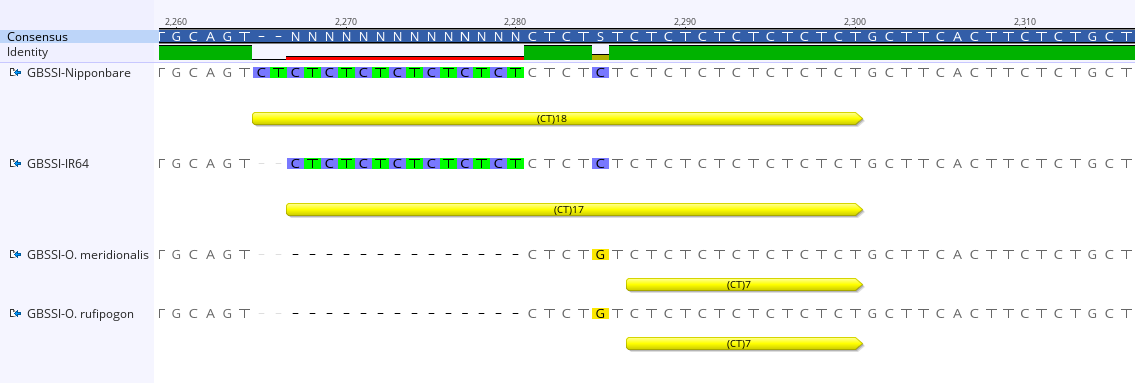


**Fig. S9** CT repeats differences in *GBSSI* among AWS, Nipponbare, and IR64

**
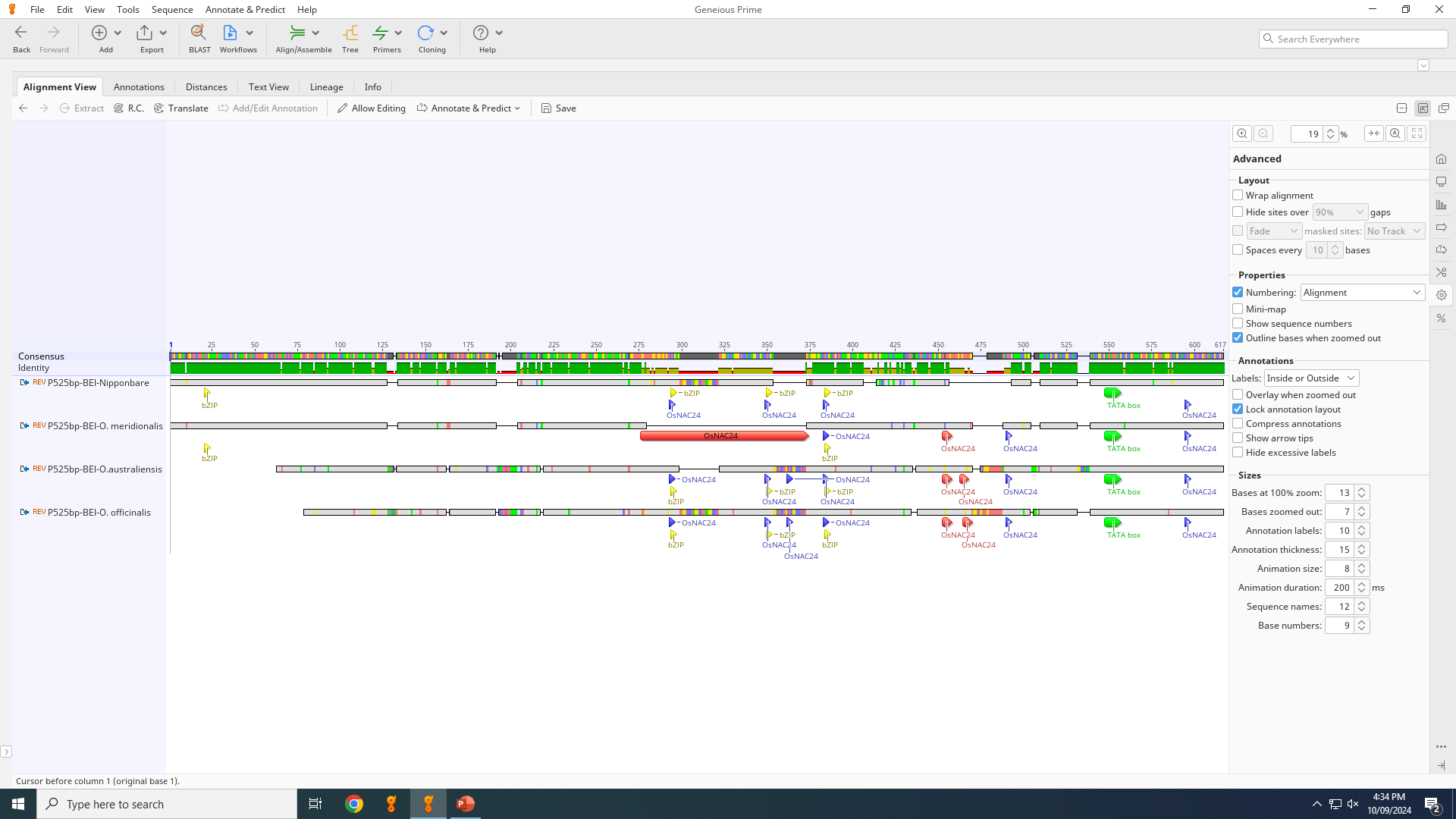
**

**Fig. S10** Promoter architecture of *BEI* among Nipponbare, *O. meridionalis*, *O. australiensis*, and *O. officinalis*. Green annotation indicates similar motifs, including TATA box and MADS box. Red and blue annotations indicate strong and weak *OsNAC24* motifs, respectively.


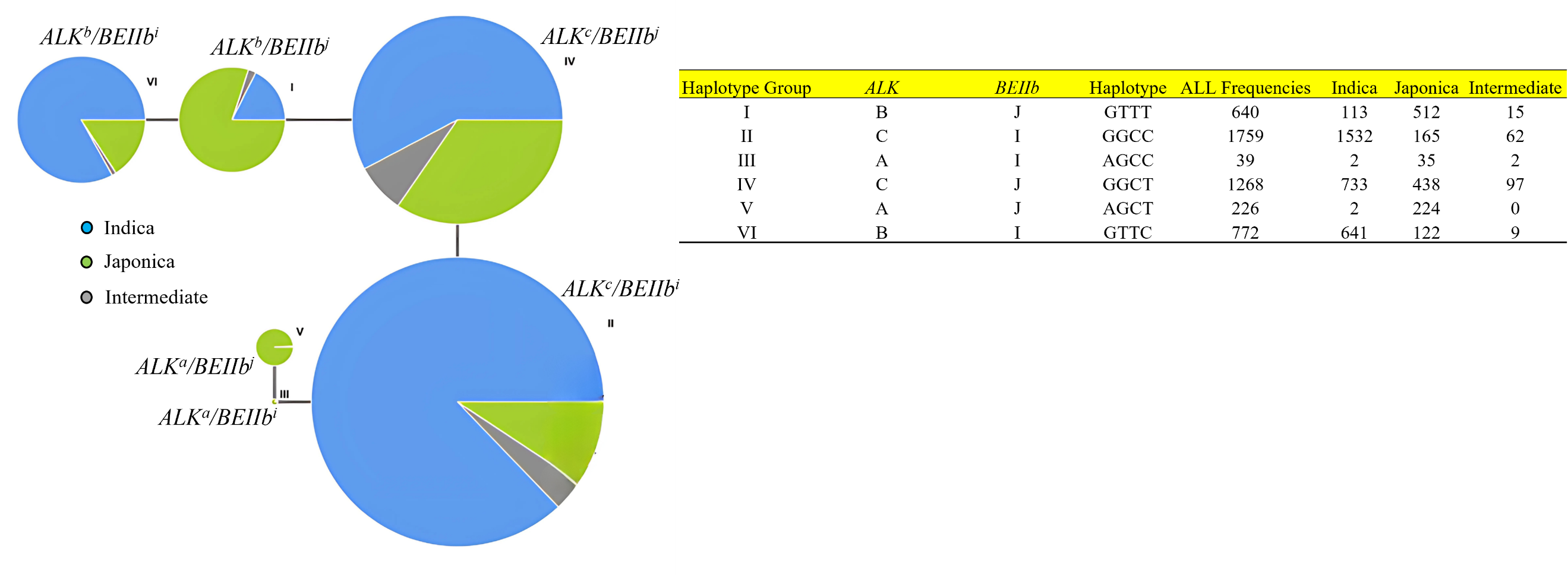


**Fig. S11** Combined haplotypes of *BEIIb* and *SSIIa* and their distribution in Asian rice accessions.

**Table S1** Selected starch-synthesis-related genes

| No | Protein/Enzyme | Genes | Chr | RAP locus | Reference |
| --- | --- | --- | --- | --- | --- |
| 1 | ADP-glucose pyrophosphorylase (large subunit) | *AGPL1/OsAPL3* | 5 | Os05g0580000 | Akihiro et al. (2005) |
| 2 |  | *AGPL2/OsAPL2* | 1 | Os01g0633100 | Akihiro et al. (2005) |
| 3 |  | *AGPL3/OsAPL1* | 3 | Os03g0735000 | Akihiro et al. (2005) |
| 4 |  | *AGPL4/OsAPL4* | 7 | Os07g0243200 | Akihiro et al. (2005) |
| 5 | ADP-glucose pyrophosphorylase (small subunit) | *AGPS1/OsAPS1* | 9 | Os09g0298200 | Akihiro et al. (2005) |
| 6 |  | *AGPS2/OsAPS2* | 8 | Os08g0345800 | Akihiro et al. (2005) |
| 7 | Starch synthase (granule bound) | *GBSSI/Waxy* | 6 | Os06g0133000 | Hirose and Terao (2004) |
| 8 |  | *GBSSII* | 7 | Os07g0412100 | Hirose and Terao (2004) |
| 9 | Starch synthase (soluble) | *SSI* | 6 | Os06g0160700 | Fujita et al. (2006) |
| 10 |  | *SSIIa* | 6 | Os06g0229800 | Unemoto et al. (2002) |
| 11 |  | *SSIIb* | 2 | Os02g0744700 |  |
| 12 |  | *SSIIc* | 10 | Os10g0437600 |  |
| 13 |  | *SSIIIa* | 8 | Os08g0191433 | Ryoo et al. (2007) |
| 14 |  | *SSIIIb* | 4 | Os04g0624600 | Ohdan et al. (2005) |
| 15 |  | *SSIVa* | 1 | Os01g0720600 | Roldan et al. (2007) |
| 16 |  | *SSIVb* | 5 | Os05g0533600 | Roldan et al. (2007) |
| 17 | Branching enzyme | *BEI* | 6 | Os06g0726400 | Sawada et al. (2018) |
| 18 |  | *BEIIa* | 4 | Os04g0409200 | Sawada et al. (2018) |
| 19 |  | *BEIIb* | 2 | Os02g0528200 | Sawada et al. (2018) |
| 20 | Debranching enzyme (Isoamylase) | *ISA1* | 8 | Os08g0520900 | Nakamura (2002) |
| 21 |  | *ISA2* | 5 | Os05g0393700 | Nakamura (2002) |
| 22 |  | *ISA3* | 9 | Os09g0469400 | Nakamura (2002); Yun et al. (2011) |
| 23 | Glucose 6-phosphate/ phosphate translocator | *GPT1* | 8 | Os08g0187800 | Toyota et al. (2006) |
| 24 |  | *GPT2* | 7 | Os07g0523600 | Toyota et al. (2006) |
| 25 | ADP-glucose transporter | *BT1* | 2 | Os02g0202400 | Li et al. (2017) |
| 26 | Disproportionating enzyme | *DPE1* | 7 | Os07g0627000 | Dong et al. (2015) |
| 27 |  | *DPE2* | 7 | Os07g0662900 | Akdogan et al. (2011) |
| 28 | Starch phosphorylase | *PHO1/PHOL* | 3 | Os03g0758100 | Satoh et al. (2008) |
| 29 |  | *PHO2/PHOH* | 1 | Os01g0851700 | Hwang et al, (2016) |
| 30 | Sucrose synthase | *OsSUS1* | 3 | Os03g0401300 | Hirose et al. (2008) |
| 31 |  | *OsSUS2* | 6 | Os06g0194900 | Hirose et al. (2008) |
| 32 |  | *OsSUS3* | 7 | Os07g0616800 | Hirose et al. (2008) |
| 33 | Transcription factor (DUF1645 domain protein) | *OsSGL* | 2 | Os02g0134200 | Liu et al. (2022) |
| 34 | Transcription factor (basic leucine zipper protein) | *OsbZIP33* | 3 | Os03g0796900 | Cai et al. (2001) |
| 35 | Transcription factor (basic leucine zipper protein) | *OsbZIP58/RISBZ1* | 7 | Os07g0182000 | Wang et al. (2013) |
| 36 | Transcription factor (basic leucine zipper protein) | *OsbZIP76* | 9 | Os09g0520400 | Niu et al. (2020) |
| 37 | Transcription factor (MADS-box) | *OsMADS1* | 3 | Os03g0215400 | Liu et al. (2023) |
| 38 | Transcription factor (MADS-box) | *OsMADS14* | 3 | Os03g0752800 | Feng et al. (2022) |
| 39 | Transcription factor (MADS-box) | *OsMADS29* | 2 | Os02g0170300 | Nayar et al. (2013) |
| 40 | Transcription factor (MADS-box) | *MADS78* | 9 | Os09g0116800 | Paul et al. (2020) |
| 41 | Transcription factor (MADS-box) | *MADS79* | 1 | Os01g0975800 | Paul et al. (2020) |
| 42 | Transcription factor (MADS-box) | *OsMADS6* | 2 | Os02g0682200 | Zhang et al. (2010) |
| 43 | Transcription factor (MADS-box) | *OsMADS7* | 8 | Os08g0531700 | Zhang et al. (2018) |
| 44 | Transcription factor (DNA-binding with one finger/DOF) | *RPBF* | 2 | Os02g0252400 | Kawakatsu et al. (2009) |
| 45 | Transcription factor (Apetala2-type) | *RSR1* | 5 | Os05g0121600 | Fu and Xue (2010) |
| 46 | Transcription factor (NAM, ATAF, and CUC) | *OsNAC20* | 1 | Os01g0104500 | Wang et al. (2020) |
| 47 | Transcription factor (NAM, ATAF, and CUC) | *OsNAC26* | 1 | Os01g0393100 | Wang et al. (2020) |
| 48 | NAC Family transcriptional activator | *OsNAP* | 3 | Os03g0327800 | Jin et al. (2023) |
| 49 | Transcription factor (DNA-binding with one finger/DOF) | *OsDOF18* | 4 | Os04g0567800 | Fang et al. (2022) |
| 50 | Transcription factor (EREBP) | *OsEBP89* | 3 | Os03g0182800 | Zhu et al. (2003); Hu et  al. (2023) |
| 51 | Prp1 protein | *Du1* | 10 | Os10g0498600 | Zeng et al. (2007) |
| 52 | Glyoxalase I (GLYI) | *FLO15* | 5 | Os05g0230900 | You et al. (2019) |
| 53 | NAD-dependent cytosolic malate dehydrogenase | *FLO16* | 10 | Os10g0478200 | Teng et al. (2019) |
| 54 | Tetratricopeptide repeat (TPR) motif-containing protein | *FLO2* | 4 | Os04g0645100 | She et al. (2010) |
| 55 | Plastidic protein | *FLO6* | 3 | Os03g0686900 | Peng et al. (2014) |
| 56 | Domain of unknown function 1338 (DUF1338) | *FLO7* | 10 | Os10g0463800 | Zhang et al. (2016) |
| 57 | UDP-glucose pyrophosphorylase | *Ugp1* | 9 | Os09g0553200 | Long et al. (2017) |
| 58 | Receptor protein kinase | *FLR1* | 3 | Os03g0333200 | Wang et al. (2021) |
| 59 | Phospholipase-like protein | *FSE1* | 8 | Os08g0110700 | Long et al (2018) |
| 60 | Aminotransferase-protein homolog | *SSG6* | 6 | Os06g0130400 | Matsushima et al. (2016) |
| 61 | Ser/Thr protein kinase | *OsCDPK1* | 3 | Os03g0128700 | Jiang et al. (2018) |
| 62 | 5′-AMP-activated protein kinase | *OsGBP* | 2 | Os02g0135900 | Wang et al. (2020) |
| 63 | Pyruvate kinase | *OsPK2* | 7 | Os07g0181000 | Cai et al. (2018) |
| 64 | Histone deacetylase | *OsSRT1* | 4 | Os04g0271000 | Zhang et al. (2016) |
| 65 | Pyrophosphate-fructose 6-phosphate 1-phosphotransferase | *PFP1* | 6 | Os06g0247500 | Chen et al. (2020) |
| 66 | Otubain-like protease | *GWC1* | 8 | Os08g0537800 | Guo et al. (2020) |
| 67 | Cell-wall invertase | *GIF1/WB1* | 4 | Os04g0413500 | Wang et al. (2008) |
| 68 | Pyruvate orthophosphate dikinase | *OsPPDKB* | 5 | Os05g0405000 | Zhang et al. (2018) |
| 69 | Glycogen synthase kinases | *OsSK41* | 3 | Os03g0841800 | Hu et al. (2023) |
| 70 | Plastidic phosphoglucomutase | *pPGM* | 10 | Os10g0189100 | Lee et al. (2016) |
| 71 | Plastidic glucose translocator | *pGlcT* | 1 | Os01g0133400 | Toyota et al. (2005) |
| 72 | NF-Y/HAP transcription factor complex | *NF-YB1* | 2 | Os02g0725900 | Feng et al. (2022) |

**Table S2** Transcript identity of 72 starch-synthesis-related genes

| No | Protein/Enzyme | Genes | Chr | RAP locus | Transcript ID |
| --- | --- | --- | --- | --- | --- |
| 1 | ADP-glucose pyrophosphorylase (large subunit) | *AGPL1/OsAPL3* | 5 | Os05g0580000 | Os05t0580000-01 |
| 2 |  | *AGPL2/OsAPL2* | 1 | Os01g0633100 | Os01t0633100-01 |
| 3 |  | *AGPL3/OsAPL1* | 3 | Os03g0735000 | Os03t0735000-01 |
| 4 |  | *AGPL4/OsAPL4* | 7 | Os07g0243200 | Os07t0243200-01 |
| 5 | ADP-glucose pyrophosphorylase (small subunit) | *AGPS1/OsAPS1* | 9 | Os09g0298200 | Os09t0298200-01 |
| 6 |  | *AGPS2/OsAPS2* | 8 | Os08g0345800 | Os08t0345800-01 |
| 7 | Starch synthase (granule bound) | *GBSSI/Waxy* | 6 | Os06g0133000 | Os06t0133000-01 |
| 8 |  | *GBSSII* | 7 | Os07g0412100 | Os07t0412100-01 |
| 9 | Starch synthase (soluble) | *SSI* | 6 | Os06g0160700 | Os06t0160700-01 |
| 10 |  | *SSIIa* | 6 | Os06g0229800 | Os06t0229800-01 |
| 11 |  | *SSIIb* | 2 | Os02g0744700 | Os02t0744700-03 |
| 12 |  | *SSIIc* | 10 | Os10g0437600 | Os10t0437600-01 |
| 13 |  | *SSIIIa* | 8 | Os08g0191433 | Os08t0191433-02 |
| 14 |  | *SSIIIb* | 4 | Os04g0624600 | Os04t0624600-01 |
| 15 |  | *SSIVa* | 1 | Os01g0720600 | Os01t0720600-02 |
| 16 |  | *SSIVb* | 5 | Os05g0533600 | Os05t0533600-01 |
| 17 | Branching enzyme | *BEI* | 6 | Os06g0726400 | Os06t0726400-01 |
| 18 |  | *BEIIa* | 4 | Os04g0409200 | Os04t0409200-01 |
| 19 |  | *BEIIb* | 2 | Os02g0528200 | Os02t0528200-01 |
| 20 | Debranching enzyme (Isoamylase) | *ISA1* | 8 | Os08g0520900 | Os08t0520900-01 |
| 21 |  | *ISA2* | 5 | Os05g0393700 | Os05t0393700-01 |
| 22 |  | *ISA3* | 9 | Os09g0469400 | Os09t0469400-03 |
| 23 | Glucose 6-phosphate/ phosphate translocator | *GPT1* | 8 | Os08g0187800 | Os08t0187800-01 |
| 24 |  | *GPT2* | 7 | Os07g0523600 | Os07t0523600-01 |
| 25 | ADP-glucose transporter | *BT1* | 2 | Os02g0202400 | Os02t0202400-01 |
| 26 | Disproportionating enzyme | *DPE1* | 7 | Os07g0627000 | Os07t0627000-01 |
| 27 |  | *DPE2* | 7 | Os07g0662900 | Os07t0662900-01 |
| 28 | Starch phosphorylase | *PHO1/PHOL* | 3 | Os03g0758100 | Os03t0758100-01 |
| 29 |  | *PHO2/PHOH* | 1 | Os01g0851700 | Os01t0851700-01 |
| 30 | Sucrose synthase | *OsSUS1* | 3 | Os03g0401300 | Os03t0401300-01 |
| 31 |  | *OsSUS2* | 6 | Os06g0194900 | Os06t0194900-01 |
| 32 |  | *OsSUS3* | 7 | Os07g0616800 | Os07t0616800-01 |
| 33 | Transcription factor (DUF1645 domain protein) | *OsSGL* | 2 | Os02g0134200 | Os02t0134200-01 |
| 34 | Transcription factor (basic leucine zipper protein) | *OsbZIP33* | 3 | Os03g0796900 | Os03t0796900-01 |
| 35 | Transcription factor (basic leucine zipper protein) | *OsbZIP58/RISBZ1* | 7 | Os07g0182000 | Os07t0182000-01 |
| 36 | Transcription factor (basic leucine zipper protein) | *OsbZIP76* | 9 | Os09g0520400 | Os09t0520400-01 |
| 37 | Transcription factor (MADS-box) | *OsMADS1* | 3 | Os03g0215400 | Os03t0215400-01 |
| 38 | Transcription factor (MADS-box) | *OsMADS14* | 3 | Os03g0752800 | Os03t0752800-02 |
| 39 | Transcription factor (MADS-box) | *OsMADS29* | 2 | Os02g0170300 | Os02t0170300-01 |
| 40 | Transcription factor (MADS-box) | *MADS78* | 9 | Os09g0116800 | Os09t0116800-01 |
| 41 | Transcription factor (MADS-box) | *MADS79* | 1 | Os01g0975800 | Os01t0975800-01 |
| 42 | Transcription factor (MADS-box) | *OsMADS6* | 2 | Os02g0682200 | Os02t0682200-01 |
| 43 | Transcription factor (MADS-box) | *OsMADS7* | 8 | Os08g0531700 | Os08t0531700-01 |
| 44 | Transcription factor (DNA-binding with one finger/DOF) | *RPBF* | 2 | Os02g0252400 | Os02t0252400-01 |
| 45 | Transcription factor (Apetala2-type) | *RSR1* | 5 | Os05g0121600 | Os05t0121600-01 |
| 46 | Transcription factor (NAM, ATAF, and CUC) | *OsNAC20* | 1 | Os01g0104500 | Os01t0104500-01 |
| 47 | Transcription factor (NAM, ATAF, and CUC) | *OsNAC26* | 1 | Os01g0393100 | Os01t0393100-02 |
| 48 | NAC Family transcriptional activator | *OsNAP* | 3 | Os03g0327800 | Os03t0327800-01 |
| 49 | Transcription factor (DNA-binding with one finger/DOF) | *OsDOF18* | 4 | Os04g0567800 | Os04t0567800-01 |
| 50 | Transcription factor (EREBP) | *OsEBP89* | 3 | Os03g0182800 | Os03t0182800-01 |
| 51 | Prp1 protein | *Du1* | 10 | Os10g0498600 | Os10t0498600-01 |
| 52 | Glyoxalase I (GLYI) | *FLO15* | 5 | Os05g0230900 | Os05t0230900-01 |
| 53 | NAD-dependent cytosolic malate dehydrogenase | *FLO16* | 10 | Os10g0478200 | Os10t0478200-01 |
| 54 | Tetratricopeptide repeat (TPR) motif-containing protein | *FLO2* | 4 | Os04g0645100 | Os04t0645100-01 |
| 55 | Plastidic protein | *FLO6* | 3 | Os03g0686900 | Os03t0686900-01 |
| 56 | Domain of unknown function 1338 (DUF1338) | *FLO7* | 10 | Os10g0463800 | Os10t0463800-01 |
| 57 | UDP-glucose pyrophosphorylase | *Ugp1* | 9 | Os09g0553200 | Os09t0553200-01 |
| 58 | Receptor protein kinase | *FLR1* | 3 | Os03g0333200 | Os03t0333200-01 |
| 59 | Phospholipase-like protein | *FSE1* | 8 | Os08g0110700 | Os08t0110700-01 |
| 60 | Aminotransferase-protein homolog | *SSG6* | 6 | Os06g0130400 | Os06t0130400-01 |
| 61 | Ser/Thr protein kinase | *OsCDPK1* | 3 | Os03g0128700 | Os03t0128700-01 |
| 62 | 5′-AMP-activated protein kinase | *OsGBP* | 2 | Os02g0135900 | Os02t0135900-01 |
| 63 | Pyruvate kinase | *OsPK2* | 7 | Os07g0181000 | Os07t0181000-05 |
| 64 | Histone deacetylase | *OsSRT1* | 4 | Os04g0271000 | Os04t0271000-01 |
| 65 | Pyrophosphate-fructose 6-phosphate 1-phosphotransferase | *PFP1* | 6 | Os06g0247500 | Os06t0247500-01 |
| 66 | Otubain-like protease | *GWC1* | 8 | Os08g0537800 | Os08t0537800-01 |
| 67 | Cell-wall invertase | *GIF1/WB1* | 4 | Os04g0413500 | Os04t0413500-01 |
| 68 | Pyruvate orthophosphate dikinase | *OsPPDKB* | 5 | Os05g0405000 | Os05t0405000-02 |
| 69 | Glycogen synthase kinases | *OsSK41* | 3 | Os03g0841800 | Os03t0841800-01 |
| 70 | Plastidic phosphoglucomutase | *pPGM* | 10 | Os10g0189100 | Os10t0189100-01 |
| 71 | Plastidic glucose translocator | *pGlcT* | 1 | Os01g0133400 | Os01t0133400-01 |
| 72 | NF-Y/HAP transcription factor complex | *NF-YB1* | 2 | Os02g0725900 | Os02t0725900-01 |

**Tabel S3** Gene classification based on expression level

| **No.** | **Expression level** | **Gene name** | **Log2 (RPKM+1) range** | **Total gene** |
| --- | --- | --- | --- | --- |
| 1 | High | *AGPS2, GBSSI, BEI, BEIIb, OsPPDKB* | 9.17-11.92 | 5 |
| 2 | upper-moderate | *AGPL1, AGPL2, AGPS1, SSI, SSIIa, SSIIIa, BT1, GPT1, Ugp1, OsSUS2, OsSUS3, PHO1, PHO2, FLO2, FLO7, FLO16, OSNAC20, OSNAC26, OsNAP, RPBF, OsMADS1* | 6.14-8.74 | 21 |
| 3 | lower-moderate | *AGPL3, GBSSII, SSIIc, SSIIIb, SSIVb, BEIIa, ISA1, ISA2, ISA3, DPE1, DPE2, pGlcT, pPGM, OsSUS1, OsMADS6, OsMADS7, OsMADS29, PFP1, OsGBP, NF-YB1, OsbZIP58, OsPK2, FSE1, FLR1, GWC1, FLO15, OsCDPK1, OsSRT1, OsSK41, Du1, SSG6, GIF1, OsEBP89* | 3.37-6.48 | 33 |
| 4 | low | *AGPL4, SSIIb, SSIVa, GPT2, OsSGL, OsbZIP33, OsbZIP76, RSR1, OsDOF18, FLO6, OsMADS14, MADS78, MADS79* | 0.01-3.06 | 13 |

**Tabel S4** 123 variants of *GBSSI* identified in 4,726 rice accessions

| **No.** | **Var ID** | **Position** | | **Variation** | **Variant position** | **Effect of variant** |
| --- | --- | --- | --- | --- | --- | --- |
|  |  | **IRGSP-1.0** | **Exon/Intron** |  |  |  |
| 1 | vg0601765639 (J) | 1765639 |  | T -> C | 5'UTR variant | - |
| 2 | vg0601765668 (J) | 1765668 |  | GTCTCTCTCT CTCTCTCTCT C -> G,GTCTCTCT CTCTCTCTCT C,GTCTC,GT CTCTC,GTCT CTCTCTCTCT CTCTCTCTCT C,GTCTCTCT CTCTCTCTC | Intron variant | - |
| 3 | vg0601765672 (J) | 1765672 |  | C -> CTCTA | Intron variant | - |
| 4 | vg0601765676 (J) | 1765676 |  | C -> A | Intron variant | - |
| 5 | vg0601765761 (J) | 1765761 | Int1-2 | T -> G | Intron variant | splice donor variant |
| 6 | vg0601765799 (J) | 1765799 |  | A -> G,C | Intron variant | - |
| 7 | vg0601765845 (J) | 1765845 |  | AGT -> A | Intron variant | - |
| 8 | vg0601765846 (J) | 1765846 |  | G -> A | Intron variant | - |
| 9 | vg0601765887 (J) | 1765887 |  | A -> AAATT,AAAT TAATT | Intron variant | - |
| 10 | vg0601765969 (J) | 1765969 |  | G -> C | Intron variant | - |
| 11 | vg0601765976 (J) | 1765976 |  | A -> G | Intron variant | - |
| 12 | vg0601765979 (J) | 1765979 |  | G -> A | Intron variant | - |
| 13 | vg0601766003 (J) | 1766003 |  | C -> T | Intron variant | - |
| 14 | vg0601766005 (J) | 1766005 |  | C -> T | Intron variant | - |
| 15 | vg0601766007 (J) | 1766007 |  | C -> T | Intron variant | - |
| 16 | vg0601766008 (J) | 1766008 |  | AG -> A | Intron variant | - |
| 17 | vg0601766012 (J) | 1766012 |  | G -> A | Intron variant | - |
| 18 | vg0601766017 (J) | 1766017 |  | C -> T | Intron variant | - |
| 19 | vg0601766018 (J) | 1766018 |  | C -> T | Intron variant | - |
| 20 | vg0601766020 (J) | 1766020 |  | A -> G | Intron variant | - |
| 21 | vg0601766029 (J) | 1766029 |  | A -> C | Intron variant | - |
| 22 | vg0601766050 (J) | 1766050 |  | C -> T | Intron variant | - |
| 23 | vg0601766054 (J) | 1766054 |  | G -> C | Intron variant | - |
| 24 | vg0601766058 (J) | 1766058 |  | TG -> T,CG | Intron variant | - |
| 25 | vg0601766071 (J) | 1766071 |  | C -> T | Intron variant | - |
| 26 | vg0601766074 (J) | 1766074 |  | A -> C,T | Intron variant | - |
| 27 | vg0601766084 (J) | 1766084 |  | T -> A,G | Intron variant | - |
| 28 | vg0601766091 (J) | 1766091 |  | G -> T | Intron variant | - |
| 29 | vg0601766100 (J) | 1766100 |  | C -> T | Intron variant | - |
| 30 | vg0601766107 (J) | 1766107 |  | TCG -> T | Intron variant | - |
| 31 | vg0601766108 (J) | 1766108 |  | C -> T | Intron variant | - |
| 32 | vg0601766110 (J) | 1766110 |  | T -> C,TAC | Intron variant | - |
| 33 | vg0601766113 (J) | 1766113 |  | C -> T,CT | Intron variant | - |
| 34 | vg0601766170 (J) | 1766170 |  | G -> GC | Intron variant | - |
| 35 | vg0601766213 (J) | 1766213 |  | T -> A | Intron variant | - |
| 36 | vg0601766308 (J) | 1766308 |  | A -> G | Intron variant | - |
| 37 | vg0601766318 (J) | 1766318 |  | CA -> C,CAA,CAAA | Intron variant | - |
| 38 | vg0601766327 (J) | 1766327 |  | A -> AT,T | Intron variant | - |
| 39 | vg0601766437 (J) | 1766437 |  | C -> T | Intron variant | - |
| 40 | vg0601766441 (J) | 1766441 |  | A -> G | Intron variant | - |
| 41 | vg0601766460 (J) | 1766460 |  | T -> C | Intron variant | - |
| 42 | vg0601766522 (J) | 1766522 |  | G -> T | Intron variant | - |
| 43 | vg0601766616 (J) | 1766616 |  | T -> C | Intron variant | - |
| 44 | vg0601766647 (J) | 1766647 |  | A -> C | Intron variant | - |
| 45 | vg0601766712 (J) | 1766712 |  | C -> T | Intron variant | - |
| 46 | vg0601766730 (J) | 1766730 |  | CT -> C,CTT,TT | Intron variant | - |
| 47 | vg0601766737 (J) | 1766737 |  | T -> C | Intron variant | - |
| 48 | vg0601766977 (J) | 1766977 | E2-57 | C -> T | Exon variant | synonymous |
| 49 | vg0601767006 (J) | 1767006 | E2-112 | G -> GCCACGGGTT CCAGGGCCTC AAGC | Exon variant | insertion |
| 50 | vg0601767052 (J) | 1767052 | E2-132 | C -> T | Exon variant | synonymous |
| 51 | vg0601767284 (J) | 1767284 |  | CTCGTCGTCG CTGACCG -> C | Intron variant | - |
| 52 | vg0601767637 (J) | 1767637 | E4-77 | A -> G | Exon variant | nonsynonymous |
| 53 | vg0601767758 (J) | 1767758 | E5-3 | T -> C | Exon variant | synonymous |
| 54 | vg0601767859 (J) | 1767859 |  | T -> G | Intron variant | - |
| 55 | vg0601767898 (J) | 1767898 |  | A -> G | Intron variant | - |
| 56 | vg0601767937 (J) | 1767937 |  | AC -> A | Intron variant | - |
| 57 | vg0601767963 (J) | 1767963 | E6-19 | A -> C |  | synonymous |
| 58 | vg0601768006 (J) | 1768006 | E6-62 | A -> C |  | nonsynonymous |
| 59 | vg0601768017 (J) | 1768017 |  | C -> T | Intron variant | - |
| 60 | vg0601768724 (J) | 1768724 | E9-202 | T -> C |  | synonymous |
| 61 | vg0601768795 (J) | 1768795 |  | A -> G | Intron variant | - |
| 62 | vg0601768821 (J) | 1768821 |  | G -> C | Intron variant | - |
| 63 | vg0601768998 (J) | 1768998 | E10-115 | C -> T |  | nonsynonymous |
| 64 | vg0601769127 (J) | 1769127 |  | CA -> C,CAA,CAAA | Intron variant | - |
| 65 | vg0601769141 (J) | 1769141 |  | G -> A | Intron variant | - |
| 66 | vg0601769154 (J) | 1769154 |  | G -> A | Intron variant | - |
| 67 | vg0601769155 (J) | 1769155 |  | G -> A | Intron variant | - |
| 68 | vg0601769158 (J) | 1769158 |  | CTAA -> C | Intron variant | - |
| 69 | vg0601769163 (J) | 1769163 |  | AAT -> A | Intron variant | - |
| 70 | vg0601769183 (J) | 1769183 |  | G -> A | Intron variant | - |
| 71 | vg0601769184 (J) | 1769184 |  | G -> A | Intron variant | - |
| 72 | vg0601769201 (J) | 1769201 |  | G -> A | Intron variant | - |
| 73 | vg0601769202 (J) | 1769202 |  | A -> G | Intron variant | - |
| 74 | vg0601769204 (J) | 1769204 |  | C -> T | Intron variant | - |
| 75 | vg0601769205 (J) | 1769205 |  | G -> A | Intron variant | - |
| 76 | vg0601769210 (J) | 1769210 |  | G -> A | Intron variant | - |
| 77 | vg0601769211 (J) | 1769211 |  | T -> C | Intron variant | - |
| 78 | vg0601769224 (J) | 1769224 |  | G -> A | Intron variant | - |
| 79 | vg0601769228 (J) | 1769228 |  | T -> C | Intron variant | - |
| 80 | vg0601769240 (J) | 1769240 |  | C -> G | Intron variant | - |
| 81 | vg0601769242 (J) | 1769242 |  | T -> C | Intron variant | - |
| 82 | vg0601769243 (J) | 1769243 |  | C -> T | Intron variant | - |
| 83 | vg0601769256 (J) | 1769256 |  | A -> G | Intron variant | - |
| 84 | vg0601769435 (J) | 1769435 | E11-132 | C -> T |  | synonymous |
| 85 | vg0601769549 (J) | 1769549 |  | CAATGCAATT GCA -> C | Intron variant | - |
| 86 | vg0601769564 (J) | 1769564 |  | TGCAAA -> CGCAAA,T | Intron variant | - |
| 87 | vg0601769643 (J) | 1769643 | E12-42 | C -> T |  | synonymous |
| 88 | vg0601769686 (J) | 1769686 | E12-85 | G -> T,A |  | nonsynonymous |
| 89 | vg0601769710 (J) | 1769710 |  | A -> G | Intron variant | - |
| 90 | vg0601769716 (J) | 1769716 |  | A -> G | Intron variant | - |
| 91 | vg0601769717 (J) | 1769717 |  | G -> T | Intron variant | - |
| 92 | vg0601769727 (J) | 1769727 |  | A -> G | Intron variant | - |
| 93 | vg0601769729 (J) | 1769729 |  | C -> T | Intron variant | - |
| 94 | vg0601769732 (J) | 1769732 |  | A -> C | Intron variant | - |
| 95 | vg0601769746 (J) | 1769746 |  | G -> A | Intron variant | - |
| 96 | vg0601769773 (J) | 1769773 |  | G -> C | Intron variant | - |
| 97 | vg0601769774 (J) | 1769774 |  | C -> T | Intron variant | - |
| 98 | vg0601769779 (J) | 1769779 |  | T -> G | Intron variant | - |
| 99 | vg0601769782 (J) | 1769782 |  | A -> G,C | Intron variant | - |
| 100 | vg0601769785 (J) | 1769785 |  | GT -> GTT,G,AT | Intron variant | - |
| 101 | vg0601769986 (J) | 1769986 |  | A -> T | Intron variant | - |
| 102 | vg0601769999 (J) | 1769999 |  | T -> C | Intron variant | - |
| 103 | vg0601770002 (J) | 1770002 |  | T -> C | Intron variant | - |
| 104 | vg0601770009 (J) | 1770009 |  | T -> A | Intron variant | - |
| 105 | vg0601770016 (J) | 1770016 |  | G -> A | Intron variant | - |
| 106 | vg0601770024 (J) | 1770024 |  | A -> G | Intron variant | - |
| 107 | vg0601770030 (J) | 1770030 |  | C -> A | Intron variant | - |
| 108 | vg0601770035 (J) | 1770035 |  | TA -> T | Intron variant | - |
| 109 | vg0601770060 (J) | 1770060 |  | C -> A | Intron variant | - |
| 110 | vg0601770065 (J) | 1770065 |  | C -> T | Intron variant | - |
| 111 | vg0601770087 (J) | 1770087 |  | AATATACTT -> A | Intron variant | - |
| 112 | vg0601770119 (J) | 1770119 |  | T -> C | Intron variant | - |
| 113 | vg0601770131 (J) | 1770131 |  | T -> G | Intron variant | - |
| 114 | vg0601770140 (J) | 1770140 |  | C -> T | Intron variant | - |
| 115 | vg0601770142 (J) | 1770142 |  | A -> G | Intron variant | - |
| 116 | vg0601770153 (J) | 1770153 |  | C -> T | Intron variant | - |
| 117 | vg0601770179 (J) | 1770179 |  | G -> A | Intron variant | - |
| 118 | vg0601770181 (J) | 1770181 |  | C -> T | Intron variant | - |
| 119 | vg0601770223 (J) | 1770223 |  | A -> T | Intron variant | - |
| 120 | vg0601770234 (J) | 1770234 |  | TC -> T | Intron variant | - |
| 121 | vg0601770385 (J) | 1770385 | E14-102 | C -> T | Exon variant | synonymous |
| 122 | vg0601770388 (J) | 1770388 | E14-105 | G -> A | Exon variant | synonymous |
| 123 | vg0601770654 (J) | 1770654 |  | T -> C | 3'UTR variant | - |

**Table S5** Variations in eight *GBSSI* haplotypes identified in selected rice accessions

| **No.** | **Haplotype** | **Genotype** | **Variant Position** | | | | | | | | | | | | | | | |
| --- | --- | --- | --- | --- | --- | --- | --- | --- | --- | --- | --- | --- | --- | --- | --- | --- | --- | --- |
|  |  |  | **I1-2** | **E2-57** | **E2-112** | **E2-132** | **E4-53** | **E4-77** | **E5-3** | **E6-19** | **E6-62** | **E9-202** | **E10-115** | **E11-132** | **E12-42** | **E12-85** | **E14-102** | **E14-105** |
| 1 | *Wx-b* | Nipponbare | T | C | - | C | G | A | T | A | A | T | C | C | C | G | C | G |
| 2 | *Wx-a* | Yikong | G | C | - | C | G | A | T | A | A | C | T | C | C | G | C | G |
| 3 | *Wx-lv* | CO_22 | G | C | - | C | G | A | T | A | A | C | C | C | C | G | C | G |
| 4 | *Wx-in* | Aomierte_168 | G | C | - | C | G | A | T | A | C | T | C | C | C | G | C | G |
| 5 | *Wx-la* | Sanbangqishiluo | T | C | - | C | G | A | T | A | C | T | C | C | C | G | C | G |
| 6 | *Wx-op* | Gajale | G | C | - | C | G | G | T | A | A | C | C | C | C | G | C | G |
| 7 | *wx* | ARC_10159 | T | C | GCCACGG  GTTCCAGG  GCCTCAAGC | C | G | A | T | A | A | T | C | C | C | G | C | G |
| 8 | *Wx-mp* | Nangeng_46 | T | C | - | C | A | A | T | A | A | T | C | C | C | G | C | G |

**Table S6** Classification of AS events

| **Genotype** | **Isoform ID** | **Isoform Length** | **No. of exon** | **Alternative splicing event** |
| --- | --- | --- | --- | --- |
| Nipponbare | PB.6833.1 | 2253 | 14 | - |
|  | PB.6833.2 | 3366 | 13 | Intron retention (Intron 1) |
|  | PB.6833.3 | 3615 | 12 | Intron retention (Intron 1 and 10) |
| *O. rufipogon* | PB.13018.1 | 1357 | 9 | Exon skipping (Exon 9, 10, 11, 12, 13) |
|  | PB.13018.2 | 2336 | 13 | Intron retention (Intron 9) + exon skipping (exon 10) |
|  | PB.13018.3 | 2327 | 13 | Intron retention (Intron 10) |
|  | PB.13018.4 | 2261 | 14 | - |
| *O. meridionalis* | PB.12139.1 | 2455 | 12 | Intron retention (Intron 8 and 11) |
|  | PB.12139.2 | 2342 | 13 | Intron retention (Intron 8 and 12) |
|  | PB.12139.3 | 2255 | 14 | - |
|  | PB.12139.4 | 2330 | 13 | Intron retention (Intron 12) |
|  | PB.12139.5 | 2269 | 14 | Intron retention (Intron 8) |
|  | PB.12139.7 | 2585 | 12 | Intron retention (Intron 8 and 10) |
|  | PB.12139.8 | 2351 | 13 | Intron retention (Intron 8) |
|  | PB.12139.9 | 2286 | 13 | Intron retention (Intron 5) |
|  | PB.12139.10 | 1657 | 11 | Exon skipping (Exon 10, 11, 12, 13, 14) |
|  | PB.12139.11 | 2543 | 13 | Intron retention (Intron 1) |
|  | PB.12139.12 | 2215 | 14 | Alternative 5' UTR |
|  | PB.12139.13 | 1914 | 13 | Exon 2 and 5' UTR skipping |

| **Genotype** | **SNP Position** | | | | | |
| --- | --- | --- | --- | --- | --- | --- |
|  | **E1-9** | **E3-1** | **E5-144** | **E14-12** | **E14-26** | **E14-104** |
| Nipponbare | TGT (C) | GTC (V) | CGC (R) | CGC (R) | CGT (R) | GGG (G) |
| *O. meridionalis* | TGC (C) | ATC (I) | CAC (H) | CGT (R) | CAT (H) | GCG (A) |
|  | syn | non-syn | non-syn | syn | non-syn | non-syn |
|  |  |  |  |  |  |  |

**Table S7** Six SNPs observed in *BEI* between Nipponbare and *O. meridionalis*

**Table S8** Haplotype classification of *BEI* in Asian rice accessions

| **Haplotype Group** | **Haplotype** | **All Frequencies** | **Indica** | **Aus** | **Japonica** | **Intermediate** |
| --- | --- | --- | --- | --- | --- | --- |
| I | CGCCAG | 3795 | 2723 | 172 | 804 | 96 |
| II | CGCTAG | 247 | 4 | 0 | 198 | 45 |
| III | CGCCGG | 533 | 3 | 1 | 495 | 34 |
| IV | CTCCAG | 123 | 16 | 96 | 1 | 10 |

**Table S9** RPKM values of putative transcription factor genes acting on *BEI*

| TF family | RAP ID | MSU ID | Gene name | RPKM value | | | | | | | | | |
| --- | --- | --- | --- | --- | --- | --- | --- | --- | --- | --- | --- | --- | --- |
|  |  |  |  | 5 DPA | | | 15 DPA | | | | 25 DPA | | |
|  |  |  |  | Nipponbare | *O. rufipogon* | *O. meridionalis* | | Nipponbare | *O. rufipogon* | *O. meridionalis* | Nipponbare | *O. rufipogon* | *O. meridionalis* |
| - | Os06g0726400 | LOC_Os06g51084 | *BEI* | 2110.25 | 1747.85 | 2344.69 | | 1326.99 | 1264.38 | 1773.10 | 446.33 | 595.67 | 1369.50 |
| NAC | Os05g0415400 | LOC_Os05g34310 | *OsNAC24* | 299.89 | 256.18 | 564.03 | | 152.28 | 139.11 | 289.33 | 13.18 | 17.60 | 71.87 |
|  | Os03g0327800 | LOC_Os03g21060 | *OsNAP* | 100.01 | 96.50 | 66.39 | | 160.63 | 161.22 | 140.86 | 99.30 | 66.38 | 91.98 |
| bZIP | Os03g0796900 | LOC_Os03g58250 | *OsbZIP33* | 6.72 | 7.57 | 5.94 | | 6.82 | 6.91 | 9.77 | 5.89 | 8.44 | 9.78 |
|  | Os07g0182000 | LOC_Os07g08420 | *OsbZIP58* | 67.24 | 58.91 | 119.29 | | 51.17 | 38.36 | 74.69 | 10.65 | 10.81 | 21.29 |
| TBP | Os12g0580300 | LOC_Os12g39070 | *OsTBP2.1* | 4.36 | 5.06 | 9.82 | | 5.17 | 7.42 | 15.29 | 3.49 | 7.46 | 7.75 |
|  | Os10g0432300 | LOC_Os10g29660 | *OsTBP2.2* | 10.66 | 11.68 | 13.96 | | 7.45 | 6.65 | 11.61 | 4.64 | 5.53 | 12.28 |
| NF-YB | Os02g0725900 | LOC_Os02g49410 | *OsNF-YB-1* | 252.21 | 126.32 | 46.12 | | 138.98 | 102.10 | 22.35 | 20.76 | 59.76 | 7.76 |
|  | Os01g0834400 | LOC_Os01g61810 | *OsNF-YB-2* | 21.08 | 21.11 | 26.07 | | 14.12 | 14.38 | 17.78 | 11.07 | 13.09 | 14.29 |
|  | Os05g0463800 | LOC_Os05g38820 | *OsNF-YB-3* | 7.81 | 9.67 | 11.56 | | 5.73 | 7.26 | 7.21 | 6.57 | 7.74 | 9.07 |
|  | Os05g0573500 | LOC_Os05g49780 | *OsNF-YB-4* | 6.60 | 4.69 | 2.12 | | 4.68 | 5.18 | 0.96 | 6.96 | 6.42 | 3.72 |
| bHLH | Os01g0915600 | LOC_Os01g68700 | *OsbHLH090* | 41.05 | 39.31 | 31.78 | | 23.99 | 20.50 | 23.32 | 14.01 | 11.64 | 16.59 |
|  | Os02g0564700 | LOC_Os02g35660 | *BHLH97* | 6.67 | 5.32 | 6.16 | | 3.90 | 3.58 | 5.17 | 2.10 | 2.91 | 2.51 |
|  | Os02g0705500 | LOC_Os02g47660 | *OsBLR1* | 3.27 | 4.33 | 4.02 | | 4.24 | 4.70 | 4.68 | 4.03 | 3.05 | 7.22 |
|  | Os03g0231950 | LOC_Os03g12940 | *OsbHLH088* | 5.96 | 7.61 | 4.86 | | 11.14 | 10.36 | 7.74 | 14.41 | 14.72 | 11.83 |
|  | Os03g0728900 | LOC_Os03g12941 | *OsbHLH084* | 5.20 | 2.70 | 1.13 | | 3.68 | 1.81 | 0.67 | 2.73 | 0.61 | 1.13 |
|  | Os03g0802900 | LOC_Os03g12942 | *OsbHLH089* | 1.60 | 0.96 | 1.93 | | 1.81 | 1.33 | 0.75 | 0.90 | 0.51 | 2.01 |
|  | Os04g0350700 | LOC_Os03g12943 | *OsbHLH093* | 0.04 | 0.18 | 0.12 | | 2.60 | 2.08 | 0.39 | 0.02 | 1.31 | 1.13 |
|  | Os06g0193400 | LOC_Os03g12944 | *OsPTF1* | 44.44 | 57.67 | 36.16 | | 36.53 | 69.54 | 48.95 | 24.71 | 51.94 | 37.68 |
|  | Os06g0275600 | LOC_Os03g12945 | *OsbHLH086* | 1.67 | 1.54 | 0.79 | | 2.09 | 2.41 | 1.15 | 1.96 | 3.80 | 1.77 |
|  | Os07g0193800 | LOC_Os03g12946 | *OsbHLH094* | 1.14 | 1.03 | 1.52 | | 1.80 | 2.06 | 0.71 | 0.63 | 0.72 | 1.78 |
|  | Os08g0487700 | LOC_Os03g12947 | *OsbHLH087* | 2.70 | 1.03 | 2.37 | | 2.70 | 1.00 | 1.40 | 1.71 | 0.72 | 1.41 |
|  | Os08g0524800 | LOC_Os03g12948 | *OsbHLH91* | 14.10 | 10.96 | 4.67 | | 8.43 | 4.07 | 2.01 | 3.11 | 1.59 | 1.61 |
|  | Os08g0536800 | LOC_Os03g12949 | *OsbHLH080* | 0.32 | 0.06 | 0.07 | | 0.40 | 0.31 | 0.11 | 0.10 | 0.11 | 0.51 |
|  | Os09g0417400 | LOC_Os03g12950 | *OsbHLH100* | 0.82 | 1.33 | 1.23 | | 2.12 | 2.66 | 1.12 | 1.24 | 0.94 | 1.62 |
|  | Os09g0474100 | LOC_Os03g12951 | *OsbHLH085* | 1.76 | 2.90 | 1.43 | | 2.33 | 2.62 | 2.41 | 3.27 | 2.73 | 2.86 |
|  | Os09g0501600 | LOC_Os03g12952 | *OsbHLH92* | 53.95 | 32.06 | 23.25 | | 38.11 | 19.61 | 19.72 | 23.28 | 9.97 | 9.89 |
|  | Os09g0510500 | LOC_Os03g12953 | *BHLH081* | 2.46 | 1.86 | 2.33 | | 2.32 | 2.30 | 1.24 | 1.31 | 0.83 | 1.36 |
|  | Os04g0429400 | LOC_Os04g35010 | *BHLH144* | 33.54 | 21.63 | 12.02 | | 25.67 | 14.75 | 7.12 | 13.30 | 4.23 | 0.97 |

| Haplotype Group | Haplotype | All Frequencies | Indica | Aus | Japonica | Intermediate |
| --- | --- | --- | --- | --- | --- | --- |
| I | CTTANTNNGCC | 2018 | 580 | 225 | 1101 | 112 |
| II | CCTANTNNGCC | 2443 | 2128 | 15 | 232 | 68 |
| III | CCNNNTNNACC | 93 | 6 | 0 | 86 | 1 |
| IV | TTNNNTNNGCC | 11 | 0 | 0 | 11 | 0 |
| V | TCCANTNNGCG | 12 | 4 | 8 | 0 | 0 |
| Total | | 4577 | 2718 | 248 | 1430 | 181 |
| SNP ID | vg0219366104, vg0219365137, vg0219361402, vg0219361396, vg0219361347, vg0219360970, vg0219360942, vg0219358908, vg0219357654, vg0219357011, vg0219356279 | | | | | |

**Table S10** Haplotype classification of *BEIIb* in Asian rice accessions

**Table S11** Haplotype classification of *SSIIa*

| Haplotype | Type | E8-733 | E8-864 | E8-865 | *SSIIa* haplotype |
| --- | --- | --- | --- | --- | --- |
| *ALK^a^* |  | ATG (Met) | GGG (Gly) | CTC (Leu) |  |
| *ALK^b^* |  | GTG (Val) | GGT (Gly) | TTC (Phe) |  |
| *ALK^c^* |  | GTG (Val) | GGG (Gly) | CTC (Leu) |  |
| Nipponbare | Japonica | ATG (Met) | GGG (Gly) | CTC (Leu) | *ALK^a^* |
| *O. rufipogon* Australia | Wild | GTG (Val) | GGG (Gly) | CTC (Leu) | *ALK^c^* |
| *O. meridionalis* | Wild | GTG (Val) | GGG (Gly) | CTC (Leu) | *ALK^c^* |
| *O. rufipogon* Asia | Wild | GTG (Val) | GGG (Gly) | CTC (Leu) | *ALK^c^* |
| *O. nivara* | Wild | GTG (Val) | GGG (Gly) | CTC (Leu) | *ALK^c^* |
| *O. barthii* | Wild | GTG (Val) | GGG (Gly) | CTC (Leu) | *ALK^c^* |
| *O. glaberrima* | African | GTG (Val) | GGG (Gly) | CTC (Leu) | *ALK^c^* |
| Kitaake | Japonica | GTG (Val) | GGT (Gly) | TTC (Phe) | *ALK^b^* |
| Kasalath | Indica | GTG (Val) | GGG (Gly) | CTC (Leu) | *ALK^c^* |
| N22 | Aus | GTG (Val) | GGG (Gly) | CTC (Leu) | *ALK^c^* |
| IR64 | Indica | GTG (Val) | GGG (Gly) | CTC (Leu) | *ALK^c^* |

**Table S12**. Combined haplotypes of *BEIIb, GBSSI*, and *SSIIa* in Asian rice accessions

| **Haplotype Group** | **Haplotype** | ***GBSSI*** | ***SSIIa*** | ***BEIIb*** | **All Frequencies** | **Indica** | **Japonica** | **Intermediate** |
| --- | --- | --- | --- | --- | --- | --- | --- | --- |
| 1 | GCGGCC | *Wx^lv^* | *ALK^c^* | *BEIIb^i^* | 730 | 571 | 110 | 49 |
| 2 | GCGTTC | *Wx^lv^* | *ALK^b^* | *BEIIb^i^* | 160 | 145 | 13 | 2 |
| 3 | GTGGCC | *Wx^a^* | *ALK^c^* | *BEIIb^i^* | 850 | 844 | 2 | 4 |
| 4 | GTGTTC | *Wx^a^* | *ALK^b^* | *BEIIb^i^* | 163 | 163 | 0 | 0 |
| 5 | TCGGCC | *Wx^b^* | *ALK^c^* | *BEIIb^i^* | 122 | 80 | 38 | 4 |
| 6 | TCAGCC | *Wx^b^* | *ALK^a^* | *BEIIb^i^* | 46 | 7 | 38 | 1 |
| 7 | TCGTTC | *Wx^b^* | *ALK^b^* | *BEIIb^i^* | 440 | 326 | 107 | 7 |
| 8 | GCGGCT | *Wx^lv^* | *ALK^c^* | *BEIIb^j^* | 860 | 435 | 336 | 89 |
| 9 | GCAGCT | *Wx^lv^* | *ALK^a^* | *BEIIb^j^* | 26 | 2 | 24 | 0 |
| 10 | GCGTTT | *Wx^lv^* | *ALK^b^* | *BEIIb^j^* | 45 | 19 | 22 | 4 |
| 11 | GTGGCT | *Wx^a^* | *ALK^c^* | *BEIIb^j^* | 274 | 265 | 1 | 8 |
| 12 | GTGTTT | *Wx^a^* | *ALK^b^* | *BEIIb^j^* | 44 | 41 | 0 | 3 |
| 13 | TCGGCT | *Wx^b^* | *ALK^c^* | *BEIIb^j^* | 92 | 33 | 56 | 3 |
| 14 | TCGTTT | *Wx^b^* | *ALK^b^* | *BEIIb^j^* | 525 | 41 | 477 | 7 |
| 15 | TCAGCT | *Wx^b^* | *ALK^a^* | *BEIIb^j^* | 226 | 1 | 225 | 0 |

**Supplementary References**

Abdullah, M., Furtado, A., Masouleh, A. K., Okemo, P., & Henry, R. J. (2024). An improved haplotype resolved genome reveals more rice genes. Tropical Plants, 3(1), 0-0. <https://doi.org/10.48130/tp-0024-0007>

Akdogan, G., Kubota, J., Kubo, A., Takaha, T., & Kitamura, S. (2011). Expression and Characterization of Rice Disproportionating Enzymes. Journal of Applied Glycoscience, 58(3), 99-105. <https://doi.org/10.5458/jag.jag.JAG-2010_026>

Akihiro, T., Mizuno, K., & Fujimura, T. (2005). Gene expression of ADP-glucose pyrophosphorylase and starch contents in rice cultured cells are cooperatively regulated by sucrose and ABA. Plant Cell Physiol, 46(6), 937-946. <https://doi.org/10.1093/pcp/pci101>

Cai, Y., Li, S., Jiao, G., Sheng, Z., Wu, Y., Shao, G., Xie, L., Peng, C., Xu, J., Tang, S., Wei, X., & Hu, P. (2018). OsPK2 encodes a plastidic pyruvate kinase involved in rice endosperm starch synthesis, compound granule formation and grain filling. Plant Biotechnol J, 16(11), 1878-1891. <https://doi.org/10.1111/pbi.12923>

Chen, C., He, B., Liu, X., Ma, X., Liu, Y., Yao, H. Y., Zhang, P., Yin, J., Wei, X., Koh, H. J., Yang, C., Xue, H. W., Fang, Z., & Qiao, Y. (2020). Pyrophosphate-fructose 6-phosphate 1-phosphotransferase (PFP1) regulates starch biosynthesis and seed development via heterotetramer formation in rice (Oryza sativa L.). Plant Biotechnol J, 18(1), 83-95. <https://doi.org/10.1111/pbi.13173>

Dong, X., Zhang, D., Liu, J., Liu, Q. Q., Liu, H., Tian, L., Jiang, L., & Qu le, Q. (2015). Plastidial Disproportionating Enzyme Participates in Starch Synthesis in Rice Endosperm by Transferring Maltooligosyl Groups from Amylose and Amylopectin to Amylopectin. Plant Physiol, 169(4), 2496-2512. <https://doi.org/10.1104/pp.15.01411>

Fang, W., Zhang, Y., Zhang, W., Gu, J., Xiong, F., An, G., & Wu, Y. (2022). Rice transcription factor OsDOF18 enlarges the starch granule size by cytokinin. Current Plant Biology, 31. <https://doi.org/10.1016/j.cpb.2022.100253>

Feng, T., Wang, L., Li, L., Liu, Y., Chong, K., Theissen, G., & Meng, Z. (2022). OsMADS14 and NF-YB1 cooperate in the direct activation of OsAGPL2 and Waxy during starch synthesis in rice endosperm. New Phytol, 234(1), 77-92. <https://doi.org/10.1111/nph.17990>

Fu, F. F., & Xue, H. W. (2010). Coexpression analysis identifies Rice Starch Regulator1, a rice AP2/EREBP family transcription factor, as a novel rice starch biosynthesis regulator. Plant Physiol, 154(2), 927-938. <https://doi.org/10.1104/pp.110.159517>

Fujita, N., Yoshida, M., Asakura, N., Ohdan, T., Miyao, A., Hirochika, H., & Nakamura, Y. (2006). Function and characterization of starch synthase I using mutants in rice. Plant Physiol, 140(3), 1070-1084. <https://doi.org/10.1104/pp.105.071845>

Furtado, A. (2014). RNA extraction from developing or mature wheat seeds. In: Henry RJ, Furtado A (eds) Cereal genomics: methods and protocols. Humana Press, Totowa, pp 23–28.

Guo, L., Chen, W., Tao, L., Hu, B., Qu, G., Tu, B., Yuan, H., Ma, B., Wang, Y., Zhu, X., Qin, P., & Li, S. (2020). GWC1 is essential for high grain quality in rice. Plant Sci, 296, 110497. <https://doi.org/10.1016/j.plantsci.2020.110497>

Hirose, T., & Terao, T. (2004). A comprehensive expression analysis of the starch synthase gene family in rice (Oryza sativa L.). Planta, 220(1), 9-16. <https://doi.org/10.1007/s00425-004-1314-6>

Hirose, T., Scofield, G. N., & Terao, T. (2008). An expression analysis profile for the entire sucrose synthase gene family in rice. Plant Science, 174(5), 534-543. <https://doi.org/10.1016/j.plantsci.2008.02.009>

Hu, Z., Niu, F., Yan, P., Wang, K., Zhang, L., Yan, Y., Zhu, Y., Dong, S., Ma, F., Lan, D., Liu, S., Xin, X., Wang, Y., Yang, J., Cao, L., Wu, S., & Luo, X. (2023). The kinase OsSK41/OsGSK5 negatively regulates amylose content in rice endosperm by affecting the interaction between OsEBP89 and OsBP5. J Integr Plant Biol. <https://doi.org/10.1111/jipb.13488>

Hwang, S.K., Koper, K., Satoh, H., Okita, T.W. (2016). Rice Endosperm Starch Phosphorylase (Pho1) Assembles with Disproportionating Enzyme (Dpe1) to Form a Protein Complex That Enhances Synthesis of Malto-oligosaccharides.Journal of Biological Chemistry, 291(38), 19994-20007. <https://doi.org/10.1074/jbc.M116.735449>

Jiang, J. Z., Kuo, C. H., Chen, B. H., Chen, M. K., Lin, C. S., & Ho, S. L. (2018). Effects of OsCDPK1 on the Structure and Physicochemical Properties of Starch in Developing Rice Seeds. Int J Mol Sci, 19(10). <https://doi.org/10.3390/ijms19103247>

Jin, S. K., Xu, L. N., Leng, Y. J., Zhang, M. Q., Yang, Q. Q., Wang, S. L., Jia, S. W., Song, T., Wang, R. A., Tao, T., Liu, Q. Q., Cai, X. L., & Gao, J. P. (2023). The OsNAC24-OsNAP protein complex activates OsGBSSI and OsSBEI expression to fine-tune starch biosynthesis in rice endosperm. Plant Biotechnol J, 21(11), 2224-2240. <https://doi.org/10.1111/pbi.14124>

Kawakatsu, T., Yamamoto, M. P., Touno, S. M., Yasuda, H., & Takaiwa, F. (2009). Compensation and interaction between RISBZ1 and RPBF during grain filling in rice. Plant J, 59(6), 908-920. <https://doi.org/10.1111/j.1365-313X.2009.03925.x>

Lee, S.K., Eom, J.S., Hwang, S.K., Shin, D., An, G., Okita, T.W., & Jeon, J.S. (2016). Plastidic phosphoglucomutase and ADP-glucose pyrophosphorylase mutants impair starch synthesis in rice pollen grains and cause male sterility. Journal of Experimental Botany, 67(18), 5557–5569. <https://doi.org/10.1093/jxb/erw324>

Li, S., Wei, X., Ren, Y., Qiu, J., Jiao, G., Guo, X., Tang, S., Wan, J., & Hu, P. (2017). OsBT1 encodes an ADP-glucose transporter involved in starch synthesis and compound granule formation in rice endosperm. Sci Rep, 7, 40124. <https://doi.org/10.1038/srep40124>

Liu, Z., Jiang, S., Jiang, L., Li, W., Tang, Y., He, W., Wang, M., Xing, J., Cui, Y., Lin, Q., Yu, F., & Wang, L. (2022). Transcription factor OsSGL is a regulator of starch synthesis and grain quality in rice. J Exp Bot, 73(11), 3417-3430. <https://doi.org/10.1093/jxb/erac068>

Liu, Z., Li, P., Yu, L., Hu, Y., Du, A., Fu, X., Wu, C., Luo, D., Hu, B., Dong, H., Jiang, H., Ma, X., Huang, W., Yang, X., Tu, S., & Li, H. (2023). OsMADS1 Regulates Grain Quality, Gene Expressions, and Regulatory Networks of Starch and Storage Protein Metabolisms in Rice. Int J Mol Sci, 24(9). <https://doi.org/10.3390/ijms24098017>

Long, W., Dong, B., Wang, Y., Pan, P., Wang, Y., Liu, L., Chen, X., Liu, X., Liu, S., Tian, Y., Chen, L., & Wan, J. (2017). FLOURY ENDOSPERM8, encoding the UDP-glucose pyrophosphorylase 1, affects the synthesis and structure of starch in rice endosperm. Journal of Plant Biology, 60(5), 513-522. <https://doi.org/10.1007/s12374-017-0066-3>

Long, W., Wang, Y., Zhu, S., Jing, W., Wang, Y., Ren, Y., Tian, Y., Liu, S., Liu, X., Chen, L., Wang, D., Zhong, M., Zhang, Y., Hu, T., Zhu, J., Hao, Y., Zhu, X., Zhang, W., Wang, C., . . . Wan, J. (2018). FLOURY SHRUNKEN ENDOSPERM1 Connects Phospholipid Metabolism and Amyloplast Development in Rice. Plant Physiol, 177(2), 698-712. <https://doi.org/10.1104/pp.17.01826>

Matsushima, R., Maekawa, M., Kusano, M., Tomita, K., Kondo, H., Nishimura, H., Crofts, N., Fujita, N., & Sakamoto, W. (2016). Amyloplast Membrane Protein SUBSTANDARD STARCH GRAIN6 Controls Starch Grain Size in Rice Endosperm. Plant Physiol, 170(3), 1445-1459. <https://doi.org/10.1104/pp.15.01811>

Nayar, S., Sharma, R., Tyagi, A. K., & Kapoor, S. (2013). Functional delineation of rice MADS29 reveals its role in embryo and endosperm development by affecting hormone homeostasis. J Exp Bot, 64(14), 4239-4253. <https://doi.org/10.1093/jxb/ert231>

Niu, B., Deng, H., Li, T., Sharma, S., Yun, Q., Li, Q., E, Z., & Chen, C. (2020). OsbZIP76 interacts with OsNF-YBs and regulates endosperm cellularization in rice (Oryza sativa). J Integr Plant Biol, 62(12), 1983-1996. <https://doi.org/10.1111/jipb.12989>

Ohdan, T., Francisco, P. B., Jr., Sawada, T., Hirose, T., Terao, T., Satoh, H., & Nakamura, Y. (2005). Expression profiling of genes involved in starch synthesis in sink and source organs of rice. J Exp Bot, 56(422), 3229-3244. <https://doi.org/10.1093/jxb/eri292>

Paul, P., Dhatt, B. K., Miller, M., Folsom, J. J., Wang, Z., Krassovskaya, I., Liu, K., Sandhu, J., Yu, H., Zhang, C., Obata, T., Staswick, P., & Walia, H. (2020). MADS78 and MADS79 Are Essential Regulators of Early Seed Development in Rice. Plant Physiol, 182(2), 933-948. <https://doi.org/10.1104/pp.19.00917>

Peng, C., Wang, Y., Liu, F., Ren, Y., Zhou, K., Lv, J., Zheng, M., Zhao, S., Zhang, L., Wang, C., Jiang, L., Zhang, X., Guo, X., Bao, Y., & Wan, J. (2014). FLOURY ENDOSPERM6 encodes a CBM48 domain-containing protein involved in compound granule formation and starch synthesis in rice endosperm. Plant J, 77(6), 917-930. <https://doi.org/10.1111/tpj.12444>

Roldan, I., Wattebled, F., Mercedes Lucas, M., Delvalle, D., Planchot, V., Jimenez, S., Perez, R., Ball, S., D'Hulst, C., & Merida, A. (2007). The phenotype of soluble starch synthase IV defective mutants of Arabidopsis thaliana suggests a novel function of elongation enzymes in the control of starch granule formation. Plant J, 49(3), 492-504. <https://doi.org/10.1111/j.1365-313X.2006.02968.x>

Ryoo, N., Yu, C., Park, C. S., Baik, M. Y., Park, I. M., Cho, M. H., Bhoo, S. H., An, G., Hahn, T. R., & Jeon, J. S. (2007). Knockout of a starch synthase gene OsSSIIIa/Flo5 causes white-core floury endosperm in rice (Oryza sativa L.). Plant Cell Rep, 26(7), 1083-1095. <https://doi.org/10.1007/s00299-007-0309-8>

Sawada, T., Itoh, M., & Nakamura, Y. (2018). Contributions of Three Starch Branching Enzyme Isozymes to the Fine Structure of Amylopectin in Rice Endosperm. Front Plant Sci, 9, 1536. <https://doi.org/10.3389/fpls.2018.01536>

She, K. C., Kusano, H., Koizumi, K., Yamakawa, H., Hakata, M., Imamura, T., Fukuda, M., Naito, N., Tsurumaki, Y., Yaeshima, M., Tsuge, T., Matsumoto, K., Kudoh, M., Itoh, E., Kikuchi, S., Kishimoto, N., Yazaki, J., Ando, T., Yano, M., . . . Shimada, H. (2010). A novel factor FLOURY ENDOSPERM2 is involved in regulation of rice grain size and starch quality. Plant Cell, 22(10), 3280-3294.

Tardaguila, M., de la Fuente, L., Marti, C., Pereira, C., Pardo-Palacios, F. J., Del Risco, H., Ferrell, M., Mellado, M., Macchietto, M., Verheggen, K., Edelmann, M., Ezkurdia, I., Vazquez, J., Tress, M., Mortazavi, A., Martens, L., Rodriguez-Navarro, S., Moreno-Manzano, V., & Conesa, A. (2018). SQANTI: extensive characterization of long-read transcript sequences for quality control in full-length transcriptome identification and quantification. Genome Res, 28(3), 396-411. <https://doi.org/10.1101/gr.222976.117>

Teng, X., Zhong, M., Zhu, X., Wang, C., Ren, Y., Wang, Y., Zhang, H., Jiang, L., Wang, D., Hao, Y., Wu, M., Zhu, J., Zhang, X., Guo, X., Wang, Y., & Wan, J. (2019). FLOURY ENDOSPERM16 encoding a NAD-dependent cytosolic malate dehydrogenase plays an important role in starch synthesis and seed development in rice. Plant Biotechnol J, 17(10), 1914-1927. <https://doi.org/10.1111/pbi.13108>

Toyota, K., Tamura, M., Ohdan, T., & Nakamura, Y. (2006). Expression profiling of starch metabolism-related plastidic translocator genes in rice. Planta, 223(2), 248-257. <https://doi.org/10.1007/s00425-005-0128-5>

Umemoto, T., Yano, M., Satoh, H. *et al.* Mapping of a gene responsible for the difference in amylopectin structure between *japonica*-type and *indica*-type rice varieties. (2002). Theor Appl Genet, 104, 1–8. <https://doi.org/10.1007/s001220200000>

Wang, J. C., Xu, H., Zhu, Y., Liu, Q. Q., & Cai, X. L. (2013). OsbZIP58, a basic leucine zipper transcription factor, regulates starch biosynthesis in rice endosperm. J Exp Bot, 64(11), 3453-3466. <https://doi.org/10.1093/jxb/ert187>

Wang, H., Zhang, Y., Sun, L., Xu, P., Tu, R., Meng, S., Wu, W., Anis, G. B., Hussain, K., Riaz, A., Chen, D., Cao, L., Cheng, S., & Shen, X. (2018). WB1, a Regulator of Endosperm Development in Rice, Is Identified by a Modified MutMap Method. Int J Mol Sci, 19(8). <https://doi.org/10.3390/ijms19082159>

Wang, J., Chen, Z., Zhang, Q., Meng, S., & Wei, C. (2020). The NAC Transcription Factors OsNAC20 and OsNAC26 Regulate Starch and Storage Protein Synthesis. Plant Physiol, 184(4), 1775-1791. <https://doi.org/10.1104/pp.20.00984>

Wang, W., Wei, X., Jiao, G., Chen, W., Wu, Y., Sheng, Z., Hu, S., Xie, L., Wang, J., Tang, S., & Hu, P. (2020). GBSS-BINDING PROTEIN, encoding a CBM48 domain-containing protein, affects rice quality and yield. J Integr Plant Biol, 62(7), 948-966. <https://doi.org/10.1111/jipb.12866>

Wang, L., Wang, D., Yang, Z., Jiang, S., Qu, J., He, W., Liu, Z., Xing, J., Ma, Y., Lin, Q., & Yu, F. (2021). Roles of FERONIA-like receptor genes in regulating grain size and quality in rice. Sci China Life Sci, 64(2), 294-310. <https://doi.org/10.1007/s11427-020-1780-x>

You, X., Zhang, W., Hu, J., Jing, R., Cai, Y., Feng, Z., Kong, F., Zhang, J., Yan, H., Chen, W., Chen, X., Ma, J., Tang, X., Wang, P., Zhu, S., Liu, L., Jiang, L., & Wan, J. (2019). FLOURY ENDOSPERM15 encodes a glyoxalase I involved in compound granule formation and starch synthesis in rice endosperm. Plant Cell Rep, 38(3), 345-359. <https://doi.org/10.1007/s00299-019-02370-9>

Yun, M.S., Umemoto, T., & Kawagoe, Y. (2011). Rice Debranching Enzyme Isoamylase3 Facilitates Starch Metabolism and Affects Plastid Morphogenesis. Plant and Cell Physiology, 52(6), 1068–1082. <https://doi.org/10.1093/pcp/pcr058>

Zhang, J., Nallamilli, B. R., Mujahid, H., & Peng, Z. (2010). OsMADS6 plays an essential role in endosperm nutrient accumulation and is subject to epigenetic regulation in rice (Oryza sativa). Plant J, 64(4), 604-617. <https://doi.org/10.1111/j.1365-313X.2010.04354.x>

Zhang, L., Ren, Y., Lu, B., Yang, C., Feng, Z., Liu, Z., Chen, J., Ma, W., Wang, Y., Yu, X., Wang, Y., Zhang, W., Wang, Y., Liu, S., Wu, F., Zhang, X., Guo, X., Bao, Y., Jiang, L., & Wan, J. (2016). FLOURY ENDOSPERM7 encodes a regulator of starch synthesis and amyloplast development essential for peripheral endosperm development in rice. J Exp Bot, 67(3), 633-647. <https://doi.org/10.1093/jxb/erv469>

Zhang, H., Xu, H., Feng, M., & Zhu, Y. (2018). Suppression of OsMADS7 in rice endosperm stabilizes amylose content under high temperature stress. Plant Biotechnol J, 16(1), 18-26. <https://doi.org/10.1111/pbi.12745>

Zhang, L., Zhao, L., Lin, L., Zhao, L., Liu, Q., & Wei, C. (2018). A Novel Mutation of OsPPDKB, Encoding Pyruvate Orthophosphate Dikinase, Affects Metabolism and Structure of Starch in the Rice Endosperm. Int J Mol Sci, 19(8). <https://doi.org/10.3390/ijms19082268>

Zeng, D., Yan, M., Wang, Y., Liu, X., Qian, Q., & Li, J. (2007). Du1, encoding a novel Prp1 protein, regulates starch biosynthesis through affecting the splicing of Wxb pre-mRNAs in rice (Oryza sativa L.). Plant Mol Biol, 65(4), 501-509. <https://doi.org/10.1007/s11103-007-9186-3>

Zhu, Y., Cai, X. L., Wang, Z. Y., & Hong, M. M. (2003). An interaction between a MYC protein and an EREBP protein is involved in transcriptional regulation of the rice Wx gene. J Biol Chem, 278(48), 47803-47811. <https://doi.org/10.1074/jbc.M302806200>
